# Molluscicidal property of symbiotic bacteria associated with entomopathogenic nematodes against *Indoplanorbis exustus* and *Radix rubiginosa*, the intermediate hosts of trematode parasites

**DOI:** 10.1016/j.parepi.2024.e00375

**Published:** 2024-08-28

**Authors:** Abdulhakam Dumidae, Chanatinart Homkeaw, Chanakan Subkrasae, Jiranun Ardpairin, Supawan Pansri, Raxsina Polseela, Ittipon Phoungpetchara, Tewarat Kumchantuek, Sarunporn Tandhavanan, Aunchalee Thanwisai, Apichat Vitta

**Affiliations:** a Department of Microbiology and Parasitology, Faculty of Medical Science, Naresuan University, Phitsanulok 65000, Thailand; b Centre of Excellence in Medical Biotechnology, Faculty of Medical Science, Naresuan University, Phitsanulok 65000, Thailand; c Center of Excellence for Biodiversity, Faculty of Sciences, Naresuan University, Phitsanulok 65000, Thailand; d Department of Anatomy, Faculty of Medical Science, Naresuan University, Phitsanulok, Thailand; e Department of Microbiology and Immunology, Faculty of Tropical Medicine, Mahidol University, Bangkok 10400, Thailand

**Keywords:** Biological control, Entomopathogenic nematodes, Intermediate host, *Photorhabdus*, *Xenorhabdus*

## Abstract

*Indoplanorbis exustus* and *Radix rubiginosa* act as intermediate hosts for veterinary and medical trematode parasites. Snail control is a strategy used to decrease the number of snails and interrupt the life cycle of parasites. The objective of this study was to evaluate the efficacy of *Xenorhabdus* and *Photorhabdus* extracts against *I. exustus* and *R. rubiginosa* in the laboratory. Ethyl acetate extracts of selected symbiotic bacteria were tested for their molluscicidal activities according to World Health Organization guidelines. Additionally, pathological changes in the snails were observed after treatment with the LC50 values under a light microscope. *Indoplanorbis exustus* and *R. rubiginosa* were susceptible to all ethyl acetate extracts of symbiotic bacteria. The lowest LC50 and LC90 at 24 h for *I. exustus* after exposure to *Photorhabdus laumondii* subsp. *laumondii* (bALN18.2_TH) extracts were 81.66 and 151.02 ppm, respectively. Similarly, the lowest LC50 and LC90 at 24 h for *R. rubiginosa* after exposure to *Photorhabdus luminescence* subsp. *akhurstii* (bAPY3.5_TH) extracts were 49.21 and 147.66 ppm, respectively. *Photorhabdus* species had more substantial molluscicidal effects than *Xenorhabdus* on these snails. The ethyl acetate extracts of these bacteria are effective when contacting the epithelial cells and foot muscle of the snails. To our knowledge, this is the first report on using *Xenorhabdus* and *Photorhabdus* extracts to evaluate molluscicidal activities. These symbiotic bacteria, *Xenorhabdus* and *Photorhabdus,* may be useful for controlling snail intermediate hosts.

## 1 Introduction

*Indoplanorbis exustus* is a freshwater snail in the class Gastropoda, family Planorbidae. It's commonly known as the Indian or Asian ramshorn snail and native to South Asia, including India, Sri Lanka, Bangladesh, and Nepal. At present, due to human activities and the transportation, it has been introduced the snail to widely distribute in tropical regions throughout South Asia, Southeast Asia, the Middle East, Africa, and the French West Indies (Liu et al., 2010; Sangwan et al., 2016; Mouahid et al., 2018; Saijuntha et al., 2021). This snail has been reported as an intermediate host for veterinary and medical trematode parasites, such as *Schistosoma indicum*, *S. nasale*, *S. spindale*, *Clinostomum giganticum* and *Echinostoma* spp. (Devkota et al., 2015; Ardpairin et al., 2022; Krailas et al., 2022). These parasites contribute to the global burden of parasitic diseases, especially schistosomiasis which is one of the most prevalent parasitic diseases that affect millions of people, particularly in tropical and subtropical regions. Chronic schistosomiasis can result in severe and long-term health complications, including liver fibrosis, bladder cancer, kidney failure, infertility, and cognitive impairment (WHO, 2023). Moreover, *I. exustus* was reported to be associated with outbreaks of cercarial dermatitis in humans in Thailand, Malaysia, Laos, and India (Palmieri et al., 1977; Ditrich et al., 1992; Narain and Mahanta, 2000; Krailas et al., 2022). Another freshwater snail, *Radix rubiginosa*, previously known as *Lymnaea rubiginosa,* is an air-breathing freshwater snail in the family Lymnaeidae. In Thailand, *R. rubiginosa* has been verified as an intermediate host for *Schistosoma incognitum* (Bunnag et al., 1983), *Fasciola gigantica* (Srihakim and Pholpark, 1991) and various echinostomes (Charoenchai et al., 1997). In addition to *I. exustus* and *R. rubiginosa* being important intermediate hosts for parasitic trematodes, these snails are also commonly found attached to aquatic plants in small ponds, pools, lakes, and rice paddy fields, including in semipermanent pools in flooding fields. In addition, *I. exustus* serves as a significant plant pest, diminishing crop vigor through seed and seedling destruction, stem damage, and leaf injury, resulting in economic losses (Singla et al., 2017; Faiz and Faiz, 2020).

Controlling snails, especially schistosoma host, can help reduce parasite transmission by lowering snail populations. Several methods have been used to control snails, including chemical, biological, mechanical and environmental controls (Zheng et al., 2021). Using chemicals remains one of the most efficient methods to control the snail. The molluscicides recommended by the World Health Organization is niclosamide and it is widely used. However, there was a report of snail resistance after prolonged niclosamide application for over 20 years (Dai et al., 2014). Despite this, concerns over toxicity and cost have prompted the development of novel synthetic molluscicides (Lu et al., 2018). In the laboratory, the efficacy of several biological controls based on plant extracts against these snails has been tested. For example, extracts from *Solanum santhocarpum*, *Piper* spp., and *Tribulus terrestris* showed potential effects on *I. exustus* (Pandey and Singh, 2009; Changbunjong et al., 2010). In addition, the extract of *Solanum nigrum* showed molluscicidal activity against *Lymnaea acuminata* (Rawani et al., 2014).

Another biological agent, entomopathogenic nematodes (EPNs), are symbiotically associated with the gram-negative bacteria *Xenorhabdus* and *Photorhabdus*. The EPNs of *Steinernema* and *Heterorhabditis* are well known to be effective biocontrol agents against several insect pests (Ruiu, 2015; Labaude and Griffin, 2018). However, the molluscicidal activity of a few EPN species has been tested. In 2014 and 2017, Tunholi et al. reported that the EPNs *Heterorhabditis indica* LPP1 and *Heterorhabditis baujardi* LPP7 can kill the snails *Bradybaena similaris* and *Lymnaea columella* (Tunholi et al., 2014, Tunholi et al., 2017). In addition, *Xenorhabdus* and *Photorhabdus* bacteria show promise as alternatives for biological pest control. They effectively target a broad spectrum of insect pests by releasing secondary toxins and metabolites directly into the insect hemocoel, leading them to septicemia and dead within 24–48 h (Bode, 2009; Chaston et al., 2011). Interestingly, the symbiotic bacteria *Xenorhabdus* and *Photorhabdus* might be able to kill the intermediate host snail. A few previous reports revealed that *Bacillus thuringiensis*, an entomopathogenic bacterium showed potential molluscicidal activity against *Biomphalaria alexandrina* (Abd El-Ghany and Abd El-Ghany, 2017) and *Monacha cartusiana* snails (Gaber et al., 2022)*.* Recently, the metabolites of *Xenorhabdus* bacteria have been evaluated and showed a great potential for controlling the slug *Arion vulgaris* (Nermuť et al., 2024). However, there have been no reports about using bacterial extracts from *Xenorhabdus* and *Photorhabdus* in controlling snails. Therefore, the objectives of this work are to evaluate the efficiency of *Xenorhabdus* and *Photorhabdus* extracts on the mortality of *I. exustus* and *R. rubiginosa* snails and to study histological changes in these snails after exposure to ethyl acetate extracts from these symbiotic bacteria.

## 2 Materials and methods

### 2.1 Collection of snails

The experiments involving invertebrate animals (snails) were approved by the Center for Animal Research at Naresuan University (Project Ethics No: NU-AQ640803). *Indoplanorbis exustus* and *R. rubiginosa* were collected from a lotus basin in Phitsanulok Province, Thailand. These snails were transported at ambient temperature and under aeration to the Department of Microbiology and Parasitology, Faculty of Medical Science, Naresuan University, Phitsanulok Province. The snails were classified based on the shell morphological characteristics described by Brandt (Brandt, 1974). They were maintained in a plastic box containing dechlorinated water. The shedding method was performed to verify that the collected snails were free of cercarial trematodes before use in the experiments.

### 2.2 Bacterial strains

Symbiotic bacteria (15 isolates) isolated from entomopathogenic nematodes collected from Thailand (Table 1) were used in the present study. These bacteria frozen in nutrient agar stock (50 % glycerol) in a − 40 °C freezer were thawed and subcultured on nutrient bromothymol blue-triphenyltetrazolium chloride agar (NBTA). The plates were then kept in the dark for 3–4 days.

**Table 1:** Symbiotic bacteria and molluscicidal activity screening using whole cell suspensions.

No.	Symbiotic bacteria	Code	Mortality of snails (%)	
			*Indoplanorbis exustus*	*Radix rubiginosa*
1	*Photorhabdus luminescens*	bAPY3.5_TH	100	100
2	*Xenorhabdus stockiae*	bAPL10.3_TH	80	100
3	*Xenorhabdus stockiae*	bAST17.4_TH	100	100
4	*Xenorhabdus stockiae*	bACR12.1_TH	100	100
5	*Xenorhabdus ehlersii*	bALN7.1_TH	100	100
6	*Photorhabdus luminescens akhustii*	bALN13.2_TH	100	100
7	*Xenorhabdus ehlersii*	bALN11.5_TH	100	100
8	*Photorhabdus luminescens laumondii*	bALN18.2_TH	100	100
9	*Photorhabdus luminescens laumondii*	bALN19.2_TH	100	100
10	*Xenorhabdus griffiniae*	bMSN3.3_TH	60	40
11	*Xenorhabdus stockiae*	bSBR31.4_TH	100	60
12	*Xenorhabdus eapokensis*	bKKN2.5_TH	20	40
13	*Xenorhabdus stockiae*	bRT25.5_TH	40	80
14	*Photorhabdus luminescens akhustii*	bSBR11.1_TH	80	60
15	*Xenorhabdus thuongxuanensis*	bKKN10.1_TH	80	60
16	Control: Distilled water	–	0	0

### 2.3 Preparation of whole-cell suspensions

A single colony on NBTA of each bacterial isolate was picked up and transferred to a 15 ml tube containing 10 ml of trypticase soy broth (TSB). The tube was then incubated at room temperature for 18–24 h with shaking at 350 rpm. To maximize the whole cell suspension, 1 ml of each bacterial culture was transferred into a 50 ml tube containing 49 ml of TSB. The tube was then placed in an incubator with shaking at 150 rpm at 28 °C for 48 h. The tube containing the culture was centrifuged at 10,000 rpm for 10 min. The supernatant was decanted, while the sediment was resuspended in 12 ml of sterile distilled water. Subsequently, this solution was used as the whole cell suspension, which was used for the screening test of molluscicidal activity.

### 2.4 Molluscicidal activity of whole cell suspension

Individual *I. exustus* and *R. rubiginosa* snails were separately placed in a 24-well microwell plate with one mollusk in each well. Five wells were tested for each bacterial code, whereas a control well was filled with 2 ml of distilled water. Two milliliters of whole cells suspended in distilled water from each bacterial isolate were added to each well. The 24-well plates were closed with loose lids to prevent snail escape. Then, the plate was left at room temperature for 24 h. To observe mortality, snails were removed from a 24-well plate. They were placed in the center of a 9 ml plate containing distilled water. If there was no movement from the center of the plate within 2 h, the snails were considered to have died.

### 2.5 Preparation of bacterial extract

Based on the screening test by using whole cell suspension, 4 isolates (*Photorhabdus luminescens* bAPY3.5_TH; *Xenorhabdus stockiae* bAST17.4_TH; *Xenorhabdus ehlersii* bALN11.5_TH and *Photorhabdus luminescens laumondii* bALN18.2_TH) were selected for extraction of their crude compounds. A single colony of each selected bacterial isolate was cultured in a flask containing 200 ml of TSB. The flask was kept in an incubator with shaking at 150 rpm at 28 °C for 72 h. Subsequently, 2 volumes of ethyl acetate were mixed with the culture. The flask was placed at room temperature for 24 h. The ethyl acetate containing crude compounds was concentrated by a rotary vacuum evaporator (Buchi, Flawil, Switzerland) in triplicate. The crude compounds from each bacterial isolate were weighed and kept at −20 °C until use.

### 2.6 Bioassay

Molluscicidal testing with the ethyl acetate extracts from symbiotic bacteria was performed according to the guidelines for laboratory and field testing of molluscicides for the control of schistosomiasis (WHO, 2019). Crude extracts from each bacterial isolate were dissolved and twofold serially diluted in dimethyl sulfoxide (DMSO). The six concentrations of each bacterial extract used were 200, 100, 50, 25, 12.5 and 6.25 ppm. The controls were distilled water, 1 % DMSO, and 1 μg/ml niclosamide. Snails that were free of cercariae and approximately 1 cm in size were selected for testing. Ten snails in a 600 ml beaker containing 500 ml of each concentration of the bacterial extracts were exposed for 24 h. After exposure to the extracts, all snails were cleaned with distilled water and transferred into a beaker containing distilled water to observe mortality at 48 and 72 h. During the experiment, snails were maintained at room temperature (25 °C) with a light:dark ratio of 12:12 and fed lettuce. Triplicates of the experiments on different dates were performed.

### 2.7 Histological technique

The LC50 of bacterial extracts for each snail was selected and used to test their toxicity to snails. Snail tissues were observed at 3 h, 6 h, 12 h, and 24 h after exposure to the bacterial extracts. Histological changes in snails treated with bacterial extracts were observed under light microscopy after staining with hematoxylin and eosin. In brief, snail specimens from the treated and control groups were fixed in Davidson's solution (Moore and Barr, 1954) for 4 days. Snail samples were decalcified in 14 % EDTA with shaking at 300 rpm for 24 h at room temperature. Subsequently, tissues of the snail samples were rinsed with distilled water, placed in tissue cassettes, and washed with 70 % ethanol 3 times for 10 min each. The tissues were dehydrated with a graded series of ethanol (70 %, 80 %, 90 %, and 100 %), infiltrated with melted paraplast, and embedded in paraffin blocks. The embedded tissues were sectioned at a thickness of 5 μm using a rotary microtome (Leica RM2235, Wetzlar, Germany). The sections were transferred onto a glass slide. Then, the sections were deparaffinized with xylene and rehydrated through a graded series of ethyl alcohol (100 %, 95 %, and 70 %) for 3 min each. The sections were then stained with hematoxylin for 5 min, washed with tap water, counterstained with eosin, and mounted in Permount. The histological alterations were observed under a light microscope (Olympus BX51, Tokyo, Japan), and photomicrographs were taken using a digital camera.

### 2.8 Statistical analysis

In this study, statistical analysis was performed using STATA version 13. Analysis of the survival rate using Kaplan–Meier survival estimates was performed based on the number of dead snails at each time point, which was converted into survival time. The log-rank test for equality of survivorship functions was used to compare the survival of snails exposed to the extracts and the negative control (DMSO). A statistically significant difference was considered when the *P* value was less than 0.05.

## 3 Results

### 3.1 Mortality of snails

*Indoplanorbis exustus* and *Radix rubiginosa* were susceptible to both whole-cell suspensions (Table 1) and ethyl acetate extracts from selected symbiotic bacteria. At 24 h after exposure to the whole-cell suspensions, the highest mortality of *I. exustus* and *R. rubiginosa* was found to be 100 % (Table 1). The whole-cell suspensions from 8 isolates of symbiotic bacteria caused 100 % mortality of both snails. In contrast, the whole-cell suspension of *Xenorhabdus eapokensis* bKKN2.5_TH caused the lowest mortality of these two snails. At 24 h after snail exposure to ethyl acetate extract, the mortality of snails was highest at 100 % at the 200 ppm concentration of all extracts as well as in 1 % niclosamide (Table 2). In contrast, distilled water and 1 % DMSO induced 0 % mortality of snails. Similarly, the mortality of *R. rubiginosa* was highest at 100 % after exposure to 200 ppm concentrations of all bacterial extracts (Table 3).

**Table 2:** Cumulative mortality of *I. exustus* after exposure to ethyl acetate extracts from bacteria.

Bacteria (code)	Concentration (ppm)	Cumulate mortality (%) ± SD		
		24 h	48 h	72 h
*Xenorhabdus stockiae* (bAST17.4_TH)	200	100 ± 0	100 ± 0	100 ± 0
	100	86.7 ± 1.53	86.7 ± 1.53	96.7 ± 0.58
	50	3.3 ± 0.58	10 ± 1.0	13.3 ± 1.53
	25	0 ± 0	3.3 ± 0.58	3.3 ± 0.58
	12.5	0 ± 0	0 ± 0	0 ± 0
	6.25	0 ± 0	0 ± 0	0 ± 0
	Distilled water	0 ± 0	0 ± 0	0 ± 0
	1 % DMSO	0 ± 0	0 ± 0	0 ± 0
	1 % Niclosamide	100 ± 0	100 ± 0	100 ± 0
*Xenorhabdus ehlersii* (bALN11.5_TH)	200	100 ± 0	100 ± 0	100 ± 0
	100	63.3 ± 3.51	90 ± 1.73	96.7 ± 0.58
	50	6.7 ± 1.15	6.7 ± 1.15	6.7 ± 1.15
	25	0 ± 0	0 ± 0	0 ± 0
	12.5	0 ± 0	0 ± 0	0 ± 0
	6.25	0 ± 0	0 ± 0	0 ± 0
	Distilled water	0 ± 0	0 ± 0	0 ± 0
	1 % DMSO	0 ± 0	0 ± 0	3.3 ± 0.58
	1 % Niclosamide	100 ± 0	100 ± 0	100 ± 0
*Photorhabdus luminescence* subsp. *akhurstii* (bAPY3.5_TH)	200	100 ± 0	100 ± 0	100 ± 0
	100	100 ± 0	100 ± 0	100 ± 0
	50	36.7 ± 4.62	43.3 ± 4.93	43.3 ± 4.93
	25	3.3 ± 5.77	33.3 ± 5.77	36.7 ± 5.51
	12.5	0 ± 0	3.33 ± 0.58	3.33 ± 0.58
	6.25	0 ± 0	3.33 ± 0.58	3.33 ± 0.58
	Distilled water	0 ± 0	0 ± 0	0 ± 0
	1 % DMSO	0 ± 0	0 ± 0	3.3 ± 0.58
	1 % Niclosamide	100 ± 0	100 ± 0	100 ± 0
*Photorhabdus laumondii* subsp. *laumondii* (bALN18.2_TH)	200	100 ± 0	100 ± 0	100 ± 0
	100	96.7 ± 0.58	96.7 ± 0.58	96.7 ± 0.58
	50	36.7 ± 4.04	43.3 ± 4.04	43.3 ± 4.04
	25	6.7 ± 1.15	6.7 ± 1.15	6.7 ± 1.15
	12.5	0 ± 0	0 ± 0	3.3 ± 0.58
	6.25	0 ± 0	0 ± 0	3.3 ± 0.58
	Distilled water	0 ± 0	0 ± 0	0 ± 0
	1 % DMSO	3.3 ± 0.58	3.3 ± 0.58	6.7 ± 1.15
	1 % Niclosamide	100 ± 0	100 ± 0	100 ± 0

**Table 3:** Cumulative mortality of *R. rubiginosa* after exposure to ethyl acetate extracts from bacteria.

Bacteria (code)	Concentration (ppm)	Cumulate mortality (%) ± SD		
		24 h	48 h	72 h
*Xenorhabdus stockiae* (bAST17.4_TH)	200	100 ± 0	100 ± 0	100 ± 0
	100	96.67 ± 0.58	100 ± 0	100 ± 0
	50	20 ± 0	26.67 ± 0.58	46.67 ± 1.15
	25	10 ± 1	23.33 ± 0.58	30 ± 1
	12.5	3.33 ± 0.58	13.33 ± 1.15	16.67 ± 0.58
	6.25	3.33 ± 0.58	6.67 ± 0.58	13.33 ± 0.58
	Distilled water	0 ± 0	0 ± 0	0 ± 0
	1 % DMSO	0 ± 0	0 ± 0	0 ± 0
	1 % Niclosamide	100 ± 0	100 ± 0	100 ± 0
*Xenorhabdus ehlersii* (bALN11.5_TH)	200	100 ± 0	100 ± 0	100 ± 0
	100	96.67 ± 0.58	100 ± 0	100 ± 0
	50	30 ± 1	33.33 ± 0.58	36.67 ± 0.58
	25	13.33 ± 0.58	16.67 ± 0.58	26.67 ± 0.58
	12.5	10 ± 1	10 ± 1	13.33 ± 0.58
	6.25	0 ± 0	0 ± 0	6.67 ± 0.58
	Distilled water	0 ± 0	0 ± 0	0 ± 0
	1 % DMSO	0 ± 0	0 ± 0	0 ± 0
	1 % Niclosamide	100 ± 0	100 ± 0	100 ± 0
*Photorhabdus luminescence* subsp. *akhurstii* (bAPY3.5_TH)	200	100 ± 0	100 ± 0	100 ± 0
	100	93.33 ± 0.58	96.67 ± 0.58	100 ± 0
	50	50 ± 2	60 ± 1.73	70 ± 1.73
	25	46.67 ± 2.08	60 ± 2.65	66.67 ± 3.21
	12.5	26.67 ± 1.53	30 ± 2	53.33 ± 2.89
	6.25	23.33 ± 1.53	26.67 ± 1.53	33.33 ± 2.08
	Distilled water	0 ± 0	0 ± 0	0 ± 0
	1 % DMSO	0 ± 0	0 ± 0	0 ± 0
	1 % Niclosamide	86.67 ± 2.31	90 ± 1.73	90 ± 1.73
*Photorhabdus laumondii* subsp. *laumondii* (bALN18.2_TH)	200	100 ± 0	100 ± 0	100 ± 0
	100	90 ± 0	93.33 ± 0.58	96.67 ± 0.58
	50	36.67 ± 0.58	60 ± 1.73	73.33 ± 0.58
	25	20 ± 0	33.33 ± 0.58	43.33 ± 0.58
	12.5	6.67 ± 0.58	36.67 ± 0.58	56.67 ± 0.58
	6.25	16.67 ± 0.58	23.33 ± 1.15	33.33 ± 1.15
	Distilled water	0 ± 0	0 ± 0	0 ± 0
	1 % DMSO	0 ± 0	0 ± 0	0 ± 0
	1 % Niclosamide	100 ± 0	100 ± 0	100 ± 0

Among the bacterial extracts, the lowest LC50 and LC90 at 24 h for *I. exustus* were 81.66 and 151.02 ppm for *P. laumondii* subsp. *laumondii* (bALN18.2_TH) extracted by ethyl acetate, respectively. Similarly, the lowest LC50 and LC90 at 24 h for *R. rubiginosa* were 49.21 and 147.66 ppm, respectively, for *P. luminescence* subsp. *akhurstii* (bAPY3.5_TH) extracted by ethyl acetate (Table 4). At 72 h after *R. rubiginosa* was exposed to *P. luminescence* subsp. *akhurstii* (bAPY3.5_TH) extracts, the lowest LC50 and LC90 were 14.50 and 80.66 ppm, respectively (Table 5).

**Table 4:** The LC50 and LC90 at 24 h after exposure to the bacterial extracts of *I. exustus* and *R. rubiginosa*.

Bacteria (code)	Lethal concentration (ppm)			
	*Indoplanorbis exustus*		*Radix rubiginosa*	
	LC50	LC90	LC50	LC90
*Xenorhabdus stockiae* (bAST17.4_TH)	94.02	162.17	85.11	155.25
*Xenorhabdus ehlersii* (bALN11.5_TH)	94.42	161.33	99.87	172.08
*Photorhabdus luminescence* subsp. *akhurstii* (bAPY3.5_TH)	100.32	156.32	49.21	147.66
*Photorhabdus laumondii* subsp. *laumondii* (bALN18.2_TH)	81.66	151.02	75.51	155.16

**Table 5:** The LC50 and LC90 at 72 h after exposure to the bacterial extracts of *I. exustus* and *R. rubiginosa*.

Bacteria (code)	Lethal concentration (ppm)			
	*Indoplanorbis exustus*		*Radix rubiginosa*	
	LC50	LC90	LC50	LC90
*Xenorhabdus stockiae* (bAST17.4_TH)	89.81	156.76	47.58	90.41
*Xenorhabdus ehlersii* (bALN11.5_TH)	92.19	158.19	52.57	94.06
*Photorhabdus luminescence* subsp. *akhurstii* (bAPY3.5_TH)	100.32	156.32	14.50	80.66
*Photorhabdus laumondii* subsp. *laumondii* (bALN18.2_TH)	80.91	151.14	21.45	86.36

At 72 h after *I. exustus* exposure to the bacterial extracts, survival analysis showed that there were statistically significant differences between the snails treated with 100 and 200 ppm of all extracts and the negative control (*P* value = 0.0000; df = 1 for all comparisons) (Tables S1-S4 and Figs. S1-S4). Similarly, survival analysis of *R. rubiginosa* was statistically significant in most treated snails between the extracts and negative control (*P* value = 0.0000; df = 1 for all comparisons) (Tables S5-S6 and Figs. S5-S8).

### 3.2 Histological changes in the snails

**Fig. 1:**
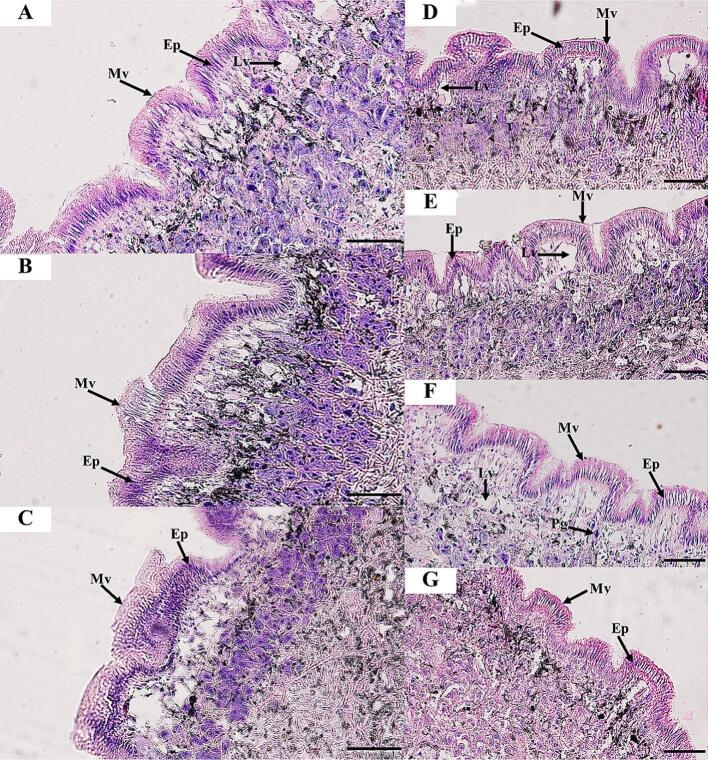
Histological structure of the *I. exustus* epidermis for 24 h under exposure to distilled water (control) (A), 1 % DMSO (B), and 1 % niclosamide (C). Snails exposed to LC50 ethyl acetate extracts from *Xenorhabdus stockiae* (bAST17.4_TH) (D), *Xenorhabdus ehlersii* (bALN11.5_TH) (E), *Photorhabdus luminescence* subsp. *akhurstii* (bAPY3.5_TH) (F), and *Photorhabdus laumondii* subsp. *laumondii* (bALN18.2_TH) extracts (G). Ep, Epidermis; Lv, lipid vacuoles; Mv, microvilli; Pg, pigment cells. Scale bar = 50 μm.

**Fig. 2:**
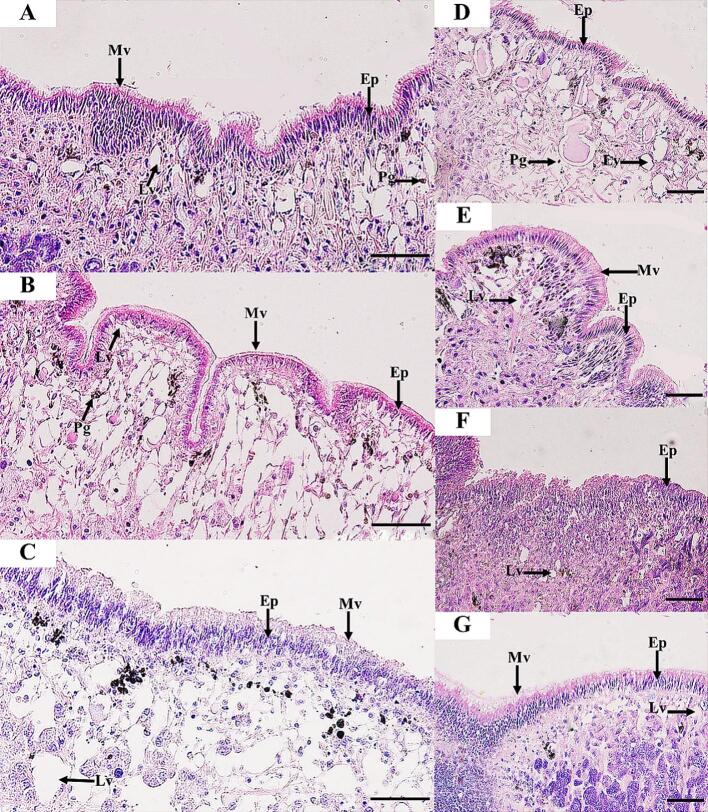
Histological structure of the *R. rubiginosa* epidermis for 24 h under exposure to distilled water (control) (A), 1 % DMSO (B), and 1 % niclosamide (C). Snails exposed to LC50 ethyl acetate extracts from *Xenorhabdus stockiae* (bAST17.4_TH) (D), *Xenorhabdus ehlersii* (bALN11.5_TH) (E), *Photorhabdus luminescence* subsp. *akhurstii* (bAPY3.5_TH) (F), and *Photorhabdus laumondii* subsp. *laumondii* (bALN18.2_TH) extracts (G). Ep, Epidermis; Lv, lipid vacuoles; Mv, microvilli; Pg, pigment cells. Scale bar = 50 μm.

**Fig. 3:**
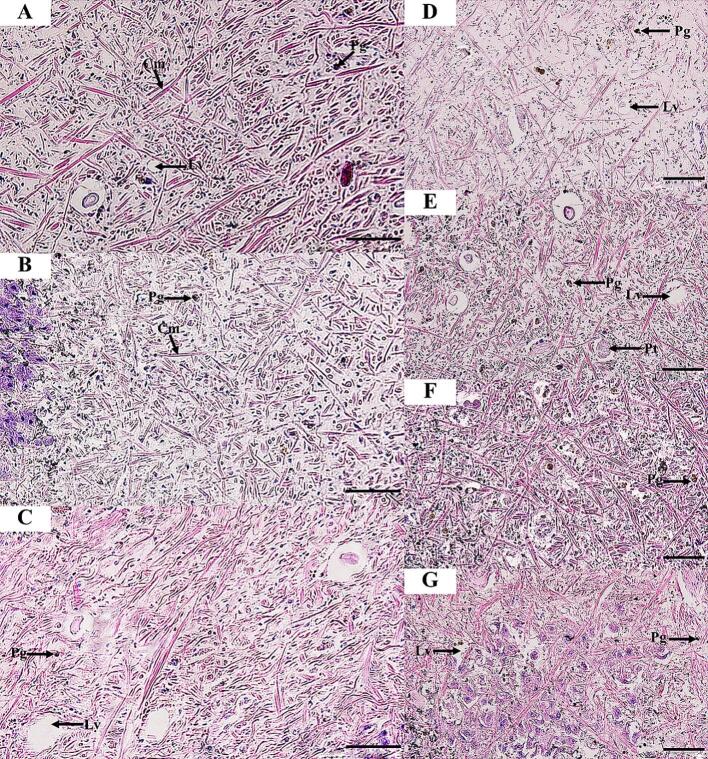
Histological structure of the *I. exustus* foot tissue for 24 h under exposure to distilled water (control) (A), 1 % DMSO (B), and 1 % niclosamide (C). Snails exposed to LC50 ethyl acetate extracts from *Xenorhabdus stockiae* (bAST17.4_TH) (D), *Xenorhabdus ehlersii* (bALN11.5_TH) (E), *Photorhabdus luminescence* subsp. *akhurstii* (bAPY3.5_TH) (F), and *Photorhabdus laumondii* subsp. *laumondii* (bALN18.2_TH) extracts (G). Cm, columnar muscle fibers; Lv, lipid vacuoles; Pg, pigment cells; Pt, protein cells. Scale bar = 50 μm.

**Fig. 4:**
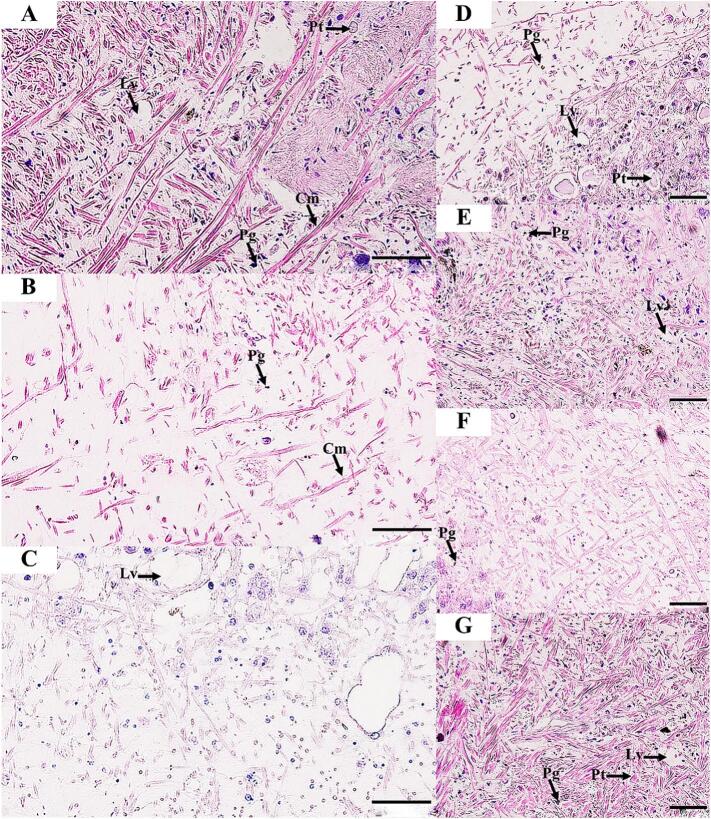
Histological structure of the *R. rubiginosa* foot tissue for 24 h under exposure to distilled water (control) (A), 1 % DMSO (B), and 1 % niclosamide (C). Snails exposed to LC50 ethyl acetate extracts from *Xenorhabdus stockiae* (bAST17.4_TH) (D), *Xenorhabdus ehlersii* (bALN11.5_TH) (E), *Photorhabdus luminescence* subsp. *akhurstii* (bAPY3.5_TH) (F), and *Photorhabdus laumondii* subsp. *laumondii* (bALN18.2_TH) extracts (G). Cm, columnar muscle fibers; Lv, lipid vacuoles; Pg, pigment cells; Pt, protein cells. Scale bar = 50 μm.

Histological features of *I. exustus* and *R. rubiginosa* exposed to bacterial extracts were observed and compared with those in normal snails. Histopathological alterations were found in the epidermis (Fig. 1, Fig. 2) and foot tissues (Fig. 3, Fig. 4) of both snails tested. In all treatments, histopathological alterations in the epidermis and foot tissues were observed after snail exposure to bacterial extract (LC50) at different times (6, 12, and 24 h). In the experimental studies, no pathological changes were observed in the epidermis and foot tissues of snails in the negative controls (distilled water and 1 % DMSO), whereas lesions on the epidermis and foot tissue of snails were observed after exposure to 1 % niclosamide (positive control) and all bacterial extracts (Fig. 1, Fig. 2, Fig. 3, Fig. 4, Figs. S9-S20).

At 3 h of exposure to the bacterial extracts, the epidermis and foot tissues of snails showed no histopathological changes. At 6 h after snail exposure to all bacterial extracts, the epidermis of *I. exustus* showed the beginning of detachment of microvilli and irregular apical surfaces and epithelial cells (Figs. S10, S16 and S13). At 12 h, desquamation continued in the epithelial cells, and lipid vacuoles were prominently found in the foot tissues, especially in the snails exposed to extracts from *P. luminescence* subsp. *akhurstii* (bAPY3.5_TH) and *X. ehlersii* (bALN11.5_TH) (Figs. S11, S14, S17, and S20). At the end of 24 h of exposure, histopathological changes were found to be milder in the epidermis, especially in *R. rubiginosa* exposed to extract from *Photorhabdus luminescence* subsp. *akhurstii* (bAPY3.5_TH), which exhibited detachment of epithelial cells (Fig. 2). In addition, an increase in the number of lipid vacuoles was also found in *R. rubiginosa* treated with the extract from *X. stockiae* (bAST17.4_TH) (Fig. 2, Fig. 4).

## 4 Discussion

The current study reported the use of *Xenorhabdus* and *Photorhabdus* bacteria as a source of crude compounds and tested their molluscicidal activity against *I. exustus* and *R. rubiginosa*. In general, *Xenorhabdus* and *Photorhabdus* are gram-negative bacilli that are symbiotically associated with steinermatid and heterorhabditid nematodes, respectively. These bacteria can produce diverse secondary metabolites, which show potential bioactivities such as antimicrobial, insecticidal, antiparasitic, and cytotoxic activities (Bode, 2009; Dreyer et al., 2018; Parihar et al., 2022). However, knowledge of the molluscicidal activity of these bacteria is limited.

In the present study, both snails *I. exustus* and *R. rubiginosa* were susceptible to all extracts from symbiotic bacteria. The mortality of both snails was highest at 100 % when these snails were treated with 200 ppm of all bacterial extracts. Moreover, the *Xenorhabdus* and *Photorhabdus* bacterial extracts exhibited the lowest LC50 and LC90 values for both snails. This agrees with the findings of several reports based on plant molluscicide activity. In an earlier study, several plant-based extracts were experimentally tested for molluscicidal activity against *I. exustus* and *Lymnaea* spp. The extract of *Agave americana* was found to be effective against all stages of *I. exustus* and *L. luteola* (Sukumaran et al., 1994). Moreover, garlic bulbs showed effective molluscicidal effects on *I. exustus* and *L. acuminata* (Singh and Singh, 1996). Additionally, other plant-based extracts, such as *Brassaia actinophylla* and *Solanum xanthocarpum*, also exhibited molluscicidal activity against *I. exustus* (Upatham et al., 1998; Changbunjong et al., 2010). Interestingly, the bacterial-based extracts used in this study were found to be more effective against *I. exustus* when compared with some plant-based extracts. In Thailand, Changbunjong and collaborators (2010) revealed that the LC50 and LC90 values of *Solanum xanthocarpum* extracts when treated with *I. exustus* were 198.00 and 236.80 ppm, respectively. These were higher than the LC50 and LC90 values of all bacterial extracts in recent study.

The choice of solvent for extraction depends on various factors such as the polarity of the compounds being extracted and the solubility of the target compound in different solvents. Solvents with a polarity value near to the polarity of the solute tend to exhibit enhanced performance (Zhang et al., 2018). Several studies have demonstrated that altering the solvent impacted the efficacy of plant extracts on snails. Sukumaran et al. (1995) reported that the n-butanol extract of *Jatropha gossypiifolia* was a more effective molluscicide than methanol extracts against *I. exustus* and L. *luteola*. In 2005, Singh and Singh reported the molluscicidal effects of the aqueous extract of latex from *Thevetia peruviana*, *Alstonia scholaris* and *Euphorbia pulcherrima* on L. *acuminata* and *I. exustus*. Therefore, continued experiments of bacterial extracts could involve altering the solvent, potentially enhancing the efficacy of these extracts against the snails. Most recently, essential oils from guava cultivars showed potential molluscicidal activity against *I. exustus* with LC50 of 3.85 to 7.71 ppm after 48 h of exposure (Luu et al., 2023). From all previous studies on molluscicidal activity mentioned herein, it can be summarized that the extracts from several species of plants have the potential to control several species of snails. The LC50 and LC90 of the extracts for controlling snails vary, possibly due to the types and volumes of the bioactive compounds in the selected plants tested. The effects of bacterial extracts on these snails in the present study might be due to the ability of bacteria to produce secondary metabolites.

In this study, the extracts of all bacteria cause the pathological change in the snail tissue, showing the lesions on the epidermis and foot tissue. Similar to the finding in a previous study of *Bithynia siamensis goniomphalos* snails, which were treated with camellia and mangosteen extracts. The results showed disruption of columnar muscle fibers and the gaps between epithelial cells and connective tissue were observed (Aukkanimart et al., 2013). The gaps between epithelial cells were also found in snail *Achatina fulica* after treated with clove oil. And the size of gaps or empty spaces within the meso-epithelial cells and muscle fibers were increased when treated with higher concentration (Parvate and Thayil, 2017). In addition, niclosamide affected the reproductive, nervous, and digestive systems of *I. exustus*, which cause vacuolated form in the cytoplasm of the neurosecretory cells, a reduction in cilia, dilation of cells and fragmentation of muscle tissue (Wangsomnuk et al., 1997). Therefore, the extracts from plants and symbiotic exerted effects on organs of the snails similar to the molluscicide. However, our study had limitations in the identification of bioactive compounds to identify specific targets in the snails. The bioactive compounds from *Xenorhabdus* and *Photorhabdus* bacteria should be isolated and identified to better understand their molluscicidal activity.

## 5 Conclusion

In summary, all bacterial extracts showed the potential to induce mortality in *I. exustus* and *R. rubiginosa* in the laboratory. *Photorhabdus* species seem to have strong molluscicidal effects on these snails. The ethyl acetate extracts from these bacteria are effective against epithelial cells and the foot muscle of the snails. These symbiotic bacteria, *Xenorhabdus* and *Photorhabdus,* may be useful for applying control strategies to snail intermediate hosts of trematode parasites.

## Funding

This study was supported by 10.13039/501100004944Naresuan University (NU), and National Science, Research and Innovation Fund (NSRF), Thailand (Grant No. R2565B047) and was partially supported by Global and Frontier Research University Fund, 10.13039/501100004944Naresuan University (Grant number R2567C003).

## Ethical standards

The experiments involving invertebrate animals (snails) were approved from the Center for Animal Research at Naresuan University (Project Ethics No: NU-AQ640803).

## Consent to participate

Not applicable.

## Consent for publication

Not applicable.

## CRediT authorship contribution statement

**Abdulhakam Dumidae:** Formal analysis, Methodology, Visualization, Writing – original draft, Writing – review & editing. **Chanatinart Homkeaw:** Methodology, Writing – original draft. **Chanakan Subkrasae:** Methodology, Writing – original draft. **Jiranun Ardpairin:** Formal analysis, Methodology, Writing – original draft, Writing – review & editing. **Supawan Pansri:** Methodology, Writing – original draft. **Raxsina Polseela:** Methodology, Visualization, Writing – original draft. **Ittipon Phoungpetchara:** Methodology, Visualization, Writing – original draft. **Tewarat Kumchantuek:** Methodology, Visualization, Writing – original draft. **Sarunporn Tandhavanan:** Formal analysis, Visualization, Writing – original draft, Writing – review & editing. **Aunchalee Thanwisai:** Methodology, Resources, Visualization, Writing – original draft, Writing – review & editing. **Apichat Vitta:** Conceptualization, Funding acquisition, Investigation, Methodology, Resources, Validation, Visualization, Writing – original draft, Writing – review & editing.

## Declaration of competing interest

The authors declare no competing interests.

## Data Availability

All relevant data used to support the finding in this study are included in this paper and the supplementary files.

## Appendix A Supplementary data

Supplementary material 1 Image 1

Supplementary material 2 Image 2

## References

[bb0005] Abd El-Ghany M.A., Abd El-Ghany M.N. (2017). Molluscicidal activity of *Bacillus thuringiensis* strains against *Biomphilaria alexandrina* snails. Beni-Suef Univ. J. Basic Appl. Sci..

[bb0010] Ardpairin J., Dumidae A., Subkrasae C., Nateeworanart S., Thanwisai A., Vitta A. (2022). Preliminary survey of larval trematodes in freshwater snails of Phitsanulok Province in lower northern Thailand. Iran. J. Parasitol..

[bb0015] Aukkanimart R., Boonmars T., Pinlaor S., Tesana S., Aunpromma S., Booyarat C., Sriraj P., Laummaunwai P., Punjaruk W. (2013). Histopathological changes in tissues of *Bithynia siamensis goniomphalos* incubated in crude extracts of camellia seed and mangosteen pericarp. Korean J. Parasitol..

[bb0020] Bode H.B. (2009). Entomopathogenic bacteria as a source of secondary metabolites. Curr. Opin. Chem. Biol..

[bb0025] Brandt R.A.M. (1974). The non-marine aquatic Mollusca of Thailand. Arch. Molluskenkd..

[bb0030] Bunnag T., Thirachandra S., Impand P., Vorasanta P., Imlarp S. (1983). *Schistosoma incognitum* and its zoonotic potential role in Phitsanulok and Phichit provinces, northern Thailand. Southeast Asian. J. Trop. Med. Public Health.

[bb0035] Changbunjong T., Wongwit W., Leemingsawat S., Tongtokit Y., Deesin V. (2010). Effect of crude extract of *Solanum xanthocarpum* against snails and mosquito larvae. Southeast Asian. J. Trop. Med. Public Health.

[bb0040] Charoenchai A., Tesana S., Pholpark M. (1997). Natural infection of trematodes in *Lymnaea* (*Radix*) *auricularia rubiginosa* in water reservoirs in Amphoe Muang, Khon Kaen Province. Southeast Asian. J. Trop. Med. Public Health.

[bb0045] Chaston J.M., Suen G., Tucker S.L., Andersen A.W., Bhasin A., Bode E., Bode H.B., Brachmann A.O., Cowles C.E., Cowles K.N., Darby C., de Léon L., Drace K., Du Z., Givaudan A., Herbert Tran E.E., Jewell K.A., Knack J.J., Krasomil-Osterfeld K.C., Kukor R., Goodrich-Blair H. (2011). The entomopathogenic bacterial endosymbionts *Xenorhabdus* and *Photorhabdus*: convergent lifestyles from divergent genomes. PLoS One.

[bb0050] Dai J., Li Y., Wang W., Xing Y., Qu G., Liang Y. (2014). Sensitivity of *Oncomelania hupensis* to niclosamide: a nation-wide survey in China. Int. J. Environ. Res. Public Health.

[bb0055] Devkota R., Brant S.V., Loker E.S. (2015). The *Schistosoma indicum* species group in Nepal: presence of a new lineage of schistosome and use of the *Indoplanorbis exustus* species complex of snail hosts. Int. J. Parasitol..

[bb0060] Ditrich O., Nasincova V., Scholz T., Giboda M. (1992). Larval stages of medically important flukes (Trematoda) from Vientiane province, Laos. Part II. Cercariae. Ann. Parasitol. Hum. Comp..

[bb0065] Dreyer J., Malan A.P., Dicks L.M.T. (2018). Bacteria of the genus *Xenorhabdus*, a novel source of bioactive compounds. Front. Microbiol..

[bb0070] Faiz A.H., Faiz L.Z. (2020). Diversity and damage assessment of snail in cultivated crops of Neelabut Bagh Azad Jammu and Kashmir (Pakistan). J. Biores. Manag..

[bb0075] Gaber O.A., Asran A.E.A., Khider F.K., El-Shahawy G., Abdel-Tawab H., Elfayoumi H.M.K. (2022). Efficacy of biopesticide Protecto (*Bacillus thuringiensis*) (BT) on certain biochemical activities and histological structures of land snail *Monacha cartusiana* (Muller, 1774). Egypt. J. Biol. Pest. Control.

[bb0080] Krailas D., Namchote S., Komsuwan J., Wongpim T., Apiraksena K., Glaubrecht M., Sonthiporn P., Sansawang C., Suwanrit S. (2022). Cercarial dermatitis outbreak caused by ruminant parasite with intermediate snail host: schistosome in Chana, South Thailand. Evol. Syst..

[bb0085] Labaude S., Griffin C.T. (2018). Transmission success of entomopathogenic nematodes used in pest control. Insects.

[bb0090] Liu L., Mondal M.M., Idris M.A., Lokman H.S., Rajapakse P.J., Satrija F., Diaz J.L., Upatham E.S., Attwood S.W. (2010). The phylogeography of *Indoplanorbis exustus* (Gastropoda: Planorbidae) in Asia. Parasit. Vectors.

[bb0095] Lu X.T., Gu Q.Y., Limpanont Y., Song L.G., Wu Z.D., Okanurak K., Lv Z.Y. (2018). Snail-borne parasitic diseases: an update on global epidemiological distribution, transmission interruption and control methods. Infect. Dis. Poverty.

[bb0100] Luu H.V.L., Nguyen H.H., Satyal P., Vo V.H., Ngo G.H., Pham V.T., Setzer W.N. (2023). Chemical composition, larvicidal and molluscicidal activity of essential oils of six guava cultivars grown in Vietnam. Plants (Basel).

[bb0105] Moore K.L., Barr M.L. (1954). Nuclear morphology, according to sex, in human tissues. Acta Anat..

[bb0110] Mouahid G., Clerissi C., Allienne J.F., Chaparro C., Al Yafae S., Mintsa-Nguema R., Ibikounlé M., Moné H. (2018). The phylogeny of the genus *Indoplanorbis* (Gastropoda, Planorbidae) from Africa and the French West Indies. Zool. Scr..

[bb0115] Narain K., Mahanta J., Bhasin M.K., Bhasin V. (2000). Man-Environment Relationship.

[bb0120] Nermuť J., Konopická J., Weijler V., Půža V. (2024). The use of *Phasmarhabditis* nematodes and metabolites of *Xenorhabdus* bacteria in slug control. Appl. Microbiol..

[bb0125] Palmieri J.R., Sullivan J.T., Ow-Yang C.K. (1977). A survey of snail hosts and larval trematodes collected by peninsular Malaysia and Singapore from 1972 to 1977. Southeast Asian. J. Trop. Med. Public Health.

[bb0130] Pandey J.K., Singh D.K. (2009). Molluscicidal activity of *Piper cubeba* Linn., *Piper longum* Linn. and *Tribulus terrestris* Linn. and their combinations against snail *Indoplanorbis exustus* Desh. Indian J. Exp. Biol..

[bb0135] Parihar R.D., Dhiman U., Bhushan A., Gupta P.K., Gupta P. (2022). *Heterorhabditis* and *Photorhabdus* symbiosis: a natural mine of bioactive compounds. Front. Microbiol..

[bb0140] Parvate Y.A., Thayil L. (2017). Toxic effect of clove oil on the survival and histology of various tissues of pestiferous land snail *Achatina fulica* (Bowdich, 1822). J. Exp. Biol. Agric. Sci..

[bb0150] Rawani A., Ghosh A., Chandra G. (2014). Laboratory evaluation of molluscicidal & mosquito larvicidal activities of leaves of *Solanum nigrum* L. Indian J. Med. Res..

[bb0155] Ruiu L. (2015). Insect pathogenic bacteria in integrated pest management. Insects.

[bb0160] Saijuntha W., Tantrawatpan C., Agatsuma T., Rajapakse R., Karunathilake K.J.K., Pilap W., Tawong W., Petney T.N., Andrews R.H. (2021). Phylogeographic genetic variation of *Indoplanorbis exustus* (Deshayes, 1834) (Gastropoda: Planorbidae) in South and Southeast Asia. One Health.

[bb0165] Sangwan A.K., Jackson B., De Glanville W., Pfeiffer D.U., Stevens K.B. (2016). Spatial analysis and identification of environmental risk factors affecting the distribution of *Indoplanorbis* and *Lymnaea* species in semi-arid and irrigated areas of Haryana, India. Parasite Epidemiol. Control.

[bb0170] Singh V.K., Singh D.K. (1996). Molluscicidal activity of pre- and post-harvest *Allium sativum* (Garlic). Biol. Agric. Hortic..

[bb0185] Singla N., Islam S., Kaur R., Singla L.D. (2017). Studies on snails inhabiting rice crop fields in Punjab state. J. Vet. Parasitol..

[bb0190] Srihakim S., Pholpark M. (1991). Problem of fascioliasis in animal husbandry in Thailand. Southeast Asian. J. Trop. Med. Public Health.

[bb0195] Sukumaran D., Parashar B.D., Rao K.M. (1994). Molluscicidal properties of *Agave americana* and *Balanites aegyptica*. Int. J. Pharm..

[bb0200] Sukumaran D., Parashar B.D., Rao K.M. (1995). Toxicity of *Jatropha gossypiifolia* and *Vaccaria pyramidata* against freshwater snails vectors of animal schistosomiasis. Fitoterapia.

[bb0205] Tunholi V.M., Monteiro C.O., Cristina da Silva L., Dolinski M., José dos Santos M.A., Rodrigues Mde L., Bittencourt V.R., Pinheiro J., Tunholi-Alves V.M. (2014). Physiological alterations in *Bradybaena similaris* (Stylommatophora: Bradybaenidae) induced by the entomopathogenic nematode *Heterorhabditis indica* (Rhabditida: Heterorhabditidae) strain LPP1. Exp. Parasitol..

[bb0210] Tunholi V.M., Lorenzoni P.O., da Silva Y.H., Tunholi-Alves V.M., Boeloni J.N., da Silva M.A., Monteiro C.O., Prata M.C.A., Pinheiro J., Martins I.V.F. (2017). Molluscicidal potential of *Heterorhabditis baujardi* (Rhabditida: Heterorhabditidae), strain LPP7, on *Lymnaea columella* (Gastropoda: Pulmonata): an alternative for biological control of fasciolosis. Acta Trop..

[bb0215] Upatham E.S., Wangsomnuk P., Kruatrachue M., Chitramvong Y.P., Reutrakul V. (1998). Acute toxicity of a plant molluscicide, *Brassaia actinophylla* on *Indoplanorbis exustus* and non-target organisms. Molluscan Res..

[bb0220] Wangsomnuk P., Upatham S.E., Kruatrachue M., Chitramvong Y., Sretarugsa P. (1997). Histological alterations in the reproductive, nervous and digestive systems of *Indoplanorbis exustus* intoxicated with molluscicides. Sci. Asia.

[bb0225] World Health Organization (WHO) (2019). https://www.who.int/publications/i/item/9789241515405.

[bb0230] World Health Organization (WHO) (2023). Schistosomiasis. https://www.who.int/newsroom/factsheets/detail/schistosomiasis.

[bb0235] Zhang Q.W., Lin L.G., Ye W.C. (2018). Techniques for extraction and isolation of natural products: a comprehensive review. Chin. Med..

[bb0240] Zheng L., Deng L., Zhong Y., Wang Y., Guo W., Fan X. (2021). Molluscicides against the snail-intermediate host of *Schistosoma*: a review. Parasitol. Res..

