# Supplementary material for: Molluscicidal property of symbiotic bacteria associated with entomopathogenic nematodes against Indoplanorbis exustus and Radix rubiginosa, the intermediate hosts of trematode parasites
Source: Parasite Epidemiol Control. 2024 Aug 28;27:e00375. doi: 10.1016/j.parepi.2024.e00375 (PMC11402155; doi:10.1016/j.parepi.2024.e00375)
Supplement: Supplementary file 1 — Supplementary material 1 [file mmc1.pdf]

# Supplementary materials Table S1-S8

**Table S1** *P*-value of log-rank test for equality of survivor functions based on comparison of the survival of *Indoplanorbis exustus* between the extracts of *Photorhabdus laumondii* subsp. *laumondii* (bALN18.2_TH) and negative control (DMSO). A statistically significant difference was considered when a *P*-value showed less than 0.05.

| | **DMSO** | **niclosamide** |
|---|---|---|
| **DW** | 0.1538 | 0.0000 |
| **DMSO** | | 0.0000 |

| | **DMSO** | **200** | **100** | **50** | **25** | **12.5** |
|---|---|---|---|---|---|---|
| **200** | 0.0000 | | | | | |
| **100** | 0.0000 | 0.3173 | | | | |
| **50** | 0.0010 | 0.0000 | 0.0000 | | | |
| **25** | 0.9929 | 0.0000 | 0.0000 | 0.0011 | | |
| **12.5** | 0.5482 | 0.0000 | 0.0000 | 0.0002 | 0.5480 | |
| **6.25** | 0.5482 | 0.0000 | 0.0000 | 0.0002 | 0.5480 | 1.0000 |

**Table S2** *P*-value of log-rank test for equality of survivor functions based on comparison of the survival of *Indoplanorbis exustus* between the extracts of *Photorhabdus luminescence* subsp. *akhurstii* (bAPY3.5_TH) and negative control (DMSO). A statistically significant difference was considered when a *P*-value showed less than 0.05.

| | **DMSO** | **niclosamide** |
|---|---|---|
| **DW** | 0.3173 | 0.0000 |
| **DMSO** | | 0.0000 |

| | **DMSO** | **200** | **100** | **50** | **25** | **12.5** |
|---|---|---|---|---|---|---|
| **200** | 0.0000 | | | | | |
| **100** | 0.0000 | 1.0000 | | | | |
| **50** | 0.0002 | 0.0000 | 0.0000 | | | |
| **25** | 0.0012 | 0.0000 | 0.0000 | 0.6031 | | |
| **12.5** | 0.9904 | 0.0000 | 0.0000 | 0.0002 | 0.0013 | |
| **6.25** | 0.9904 | 0.0000 | 0.0000 | 0.0002 | 0.0013 | 1.0000 |

**Table S3** *P*-value of log-rank test for equality of survivor functions based on comparison of the survival of *Indoplanorbis exustus* between the extracts of *Xenorhabdus ehlersii* (bALN11.5_TH) and negative control (DMSO). A statistically significant difference was considered when a *P*-value showed less than 0.05.

| | DMSO | niclosamide |
|---|---|---|
| **DW** | 0.3173 | 0.0000 |
| **DMSO** | | 0.0000 |

| | DMSO | 200 | 100 | 50 | 25 | 12.5 |
|---|---|---|---|---|---|---|
| **200** | 0.0000 | | | | | |
| **100** | 0.0000 | 0.0003 | | | | |
| **50** | 0.5480 | 0.0000 | 0.0000 | | | |
| **25** | 0.3173 | 0.0000 | 0.0000 | 0.1538 | | |
| **12.5** | 0.3173 | 0.0000 | 0.0000 | 0.1538 | na | |
| **6.25** | 0.3173 | 0.0000 | 0.0000 | 0.1538 | na | na |

**Table S4** *P*-value of log-rank test for equality of survivor functions based on comparison of the survival of *Indoplanorbis exustus* between the extracts of *Xenorhabdus stockiae* (bAST17.4_TH) and negative control (DMSO). A statistically significant difference was considered when a *P*-value showed less than 0.05.

| | DMSO | niclosamide |
|---|---|---|
| **DW** | na | 0.0000 |
| **DMSO** | | 0.0000 |

| | DMSO | 200 | 100 | 50 | 25 | 12.5 |
|---|---|---|---|---|---|---|
| **200** | 0.0000 | | | | | |
| **100** | 0.0000 | 0.0401 | | | | |
| **50** | 0.0401 | 0.0000 | 0.0000 | | | |
| **25** | 0.3173 | 0.0000 | 0.0000 | 0.1630 | | |
| **12.5** | na | 0.0000 | 0.0000 | 0.0401 | 0.3173 | |
| **6.25** | na | 0.0000 | 0.0000 | 0.0401 | 0.3173 | na |

**Table S5** *P*-value of log-rank test for equality of survivor functions based on comparison of the survival of *Radix rubiginosa* between the extracts of *Photorhabdus laumondii* subsp. *laumondii* (bALN18.2_TH) and negative control (DMSO). A statistically significant difference was considered when a *P*-value showed less than 0.05.

| | DMSO | niclosamide |
|---|---|---|
| **DW** | na | 0.0000 |
| **DMSO** | | 0.0000 |

| | DMSO | 200 | 100 | 50 | 25 | 12.5 |
|---|---|---|---|---|---|---|
| **200** | 0.0000 | | | | | |
| **100** | 0.0000 | 0.0780 | | | | |
| **50** | 0.0000 | 0.0000 | 0.0002 | | | |
| **25** | 0.0000 | 0.0000 | 0.0000 | 0.0098 | | |
| **12.5** | 0.0000 | 0.0000 | 0.0000 | 0.0282 | 0.5099 | |
| **6.25** | 0.0006 | 0.0000 | 0.0000 | 0.0008 | 0.4258 | 0.1257 |

**Table S6** *P*-value of log-rank test for equality of survivor functions based on comparison of the survival of *Radix rubiginosa* between the extracts of *Photorhabdus luminescence* subsp. *akhurstii* (bAPY3.5_TH) and negative control (DMSO). A statistically significant difference was considered when a *P*-value showed less than 0.05.

| | DMSO | niclosamide |
|---|---|---|
| **DW** | na | 0.0000 |
| **DMSO** | | 0.0000 |

| | DMSO | 200 | 100 | 50 | 25 | 12.5 |
|---|---|---|---|---|---|---|
| **200** | 0.0000 | | | | | |
| **100** | 0.0000 | 0.1538 | | | | |
| **50** | 0.0000 | 0.0000 | 0.0001 | | | |
| **25** | 0.0000 | 0.0000 | 0.0000 | 0.8062 | | |
| **12.5** | 0.0000 | 0.0000 | 0.0000 | 0.0872 | 0.1455 | |
| **6.25** | 0.0006 | 0.0000 | 0.0000 | 0.0043 | 0.0093 | 0.1690 |

**Table S7** *P*-value of log-rank test for equality of survivor functions based on comparison of the survival of *Radix rubiginosa* between the extracts of *Xenorhabdus ehlersii* (bALN11.5_TH) and negative control (DMSO). A statistically significant difference was considered when a *P*-value showed less than 0.05.

| | **DMSO** | **niclosamide** |
|---|---|---|
| **DW** | na | 0.0000 |
| **DMSO** | | 0.0000 |

| | **DMSO** | **200** | **100** | **50** | **25** | **12.5** |
|---|---|---|---|---|---|---|
| **200** | 0.0000 | | | | | |
| **100** | 0.0000 | 0.3173 | | | | |
| **50** | 0.0003 | 0.0000 | 0.0000 | | | |
| **25** | 0.0026 | 0.0000 | 0.0000 | 0.3601 | | |
| **12.5** | 0.0400 | 0.0000 | 0.0000 | 0.0370 | 0.2093 | |
| **6.25** | 0.1538 | 0.0000 | 0.0000 | 0.0040 | 0.0331 | 0.3695 |

**Table S8** *P*-value of log-rank test for equality of survivor functions based on comparison of the survival of *Radix rubiginosa* between the extracts of *Xenorhabdus stockiae* (bAST17.4_TH) and negative control (DMSO). A statistically significant difference was considered when a *P*-value showed less than 0.05.

| | **DMSO** | **niclosamide** |
|---|---|---|
| **DW** | na | 0.0000 |
| **DMSO** | | 0.0000 |

| | **DMSO** | **200** | **100** | **50** | **25** | **12.5** |
|---|---|---|---|---|---|---|
| **200** | 0.0000 | | | | | |
| **100** | 0.0000 | 0.3173 | | | | |
| **50** | 0.0000 | 0.0000 | 0.0000 | | | |
| **25** | 0.0012 | 0.0000 | 0.0000 | 0.2576 | | |
| **12.5** | 0.0205 | 0.0000 | 0.0000 | 0.0200 | 0.2168 | |
| **6.25** | 0.0401 | 0.0000 | 0.0000 | 0.0071 | 0.1100 | 0.7054 |

**Supplementary data Figure S1-S8**

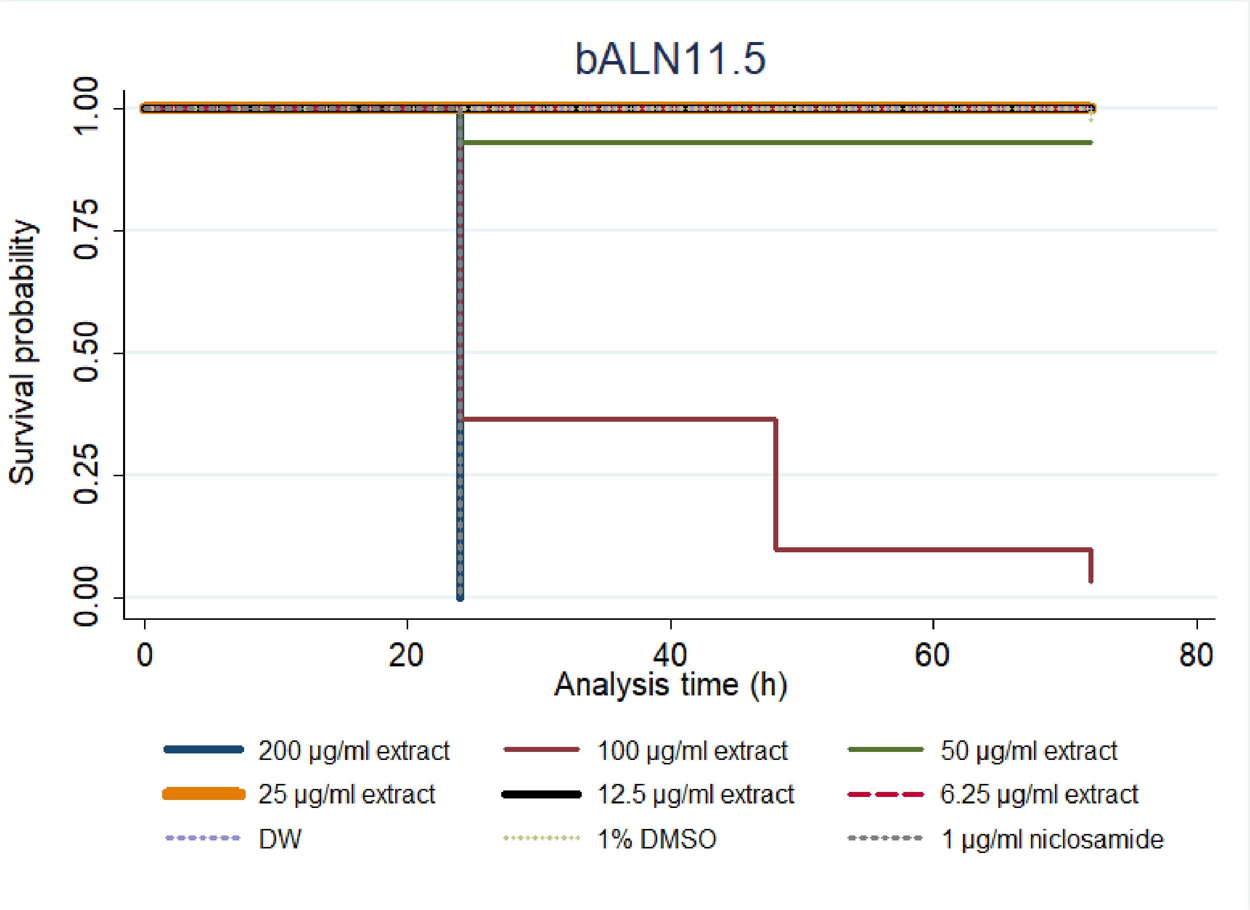


**Fig. S1** Kaplan–Meier overall survival curve comparing the mortality rates of *Indoplanorbis exustus* after exposure to the extract of *Xenorhabdus ehlersii* (bALN11.5_TH)

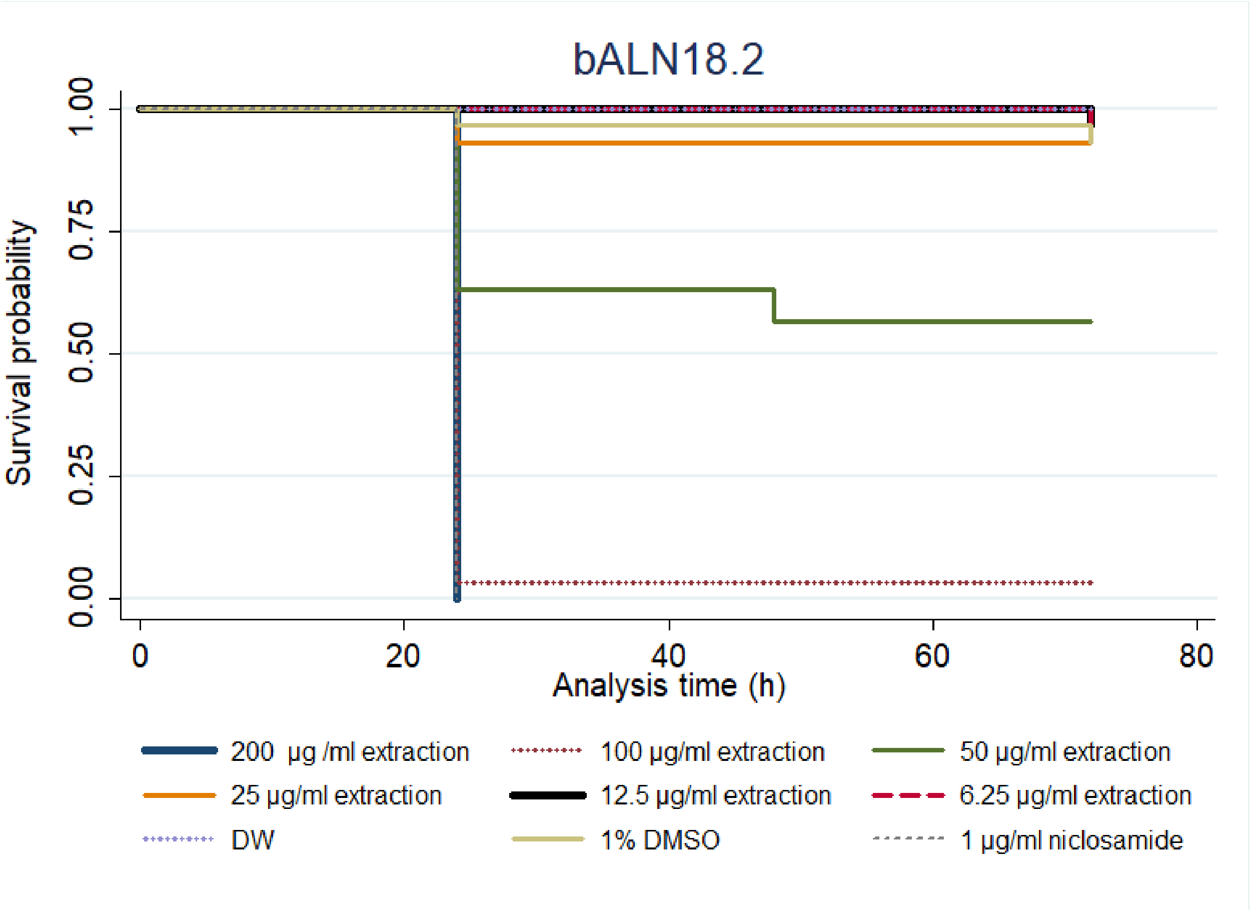


**Fig. S2** Kaplan–Meier overall survival curve comparing the mortality rates of *Indoplanorbis exustus* after exposure to the extract of *Photorhabdus laumondii* subsp. *laumondii* (bALN18.2_TH)

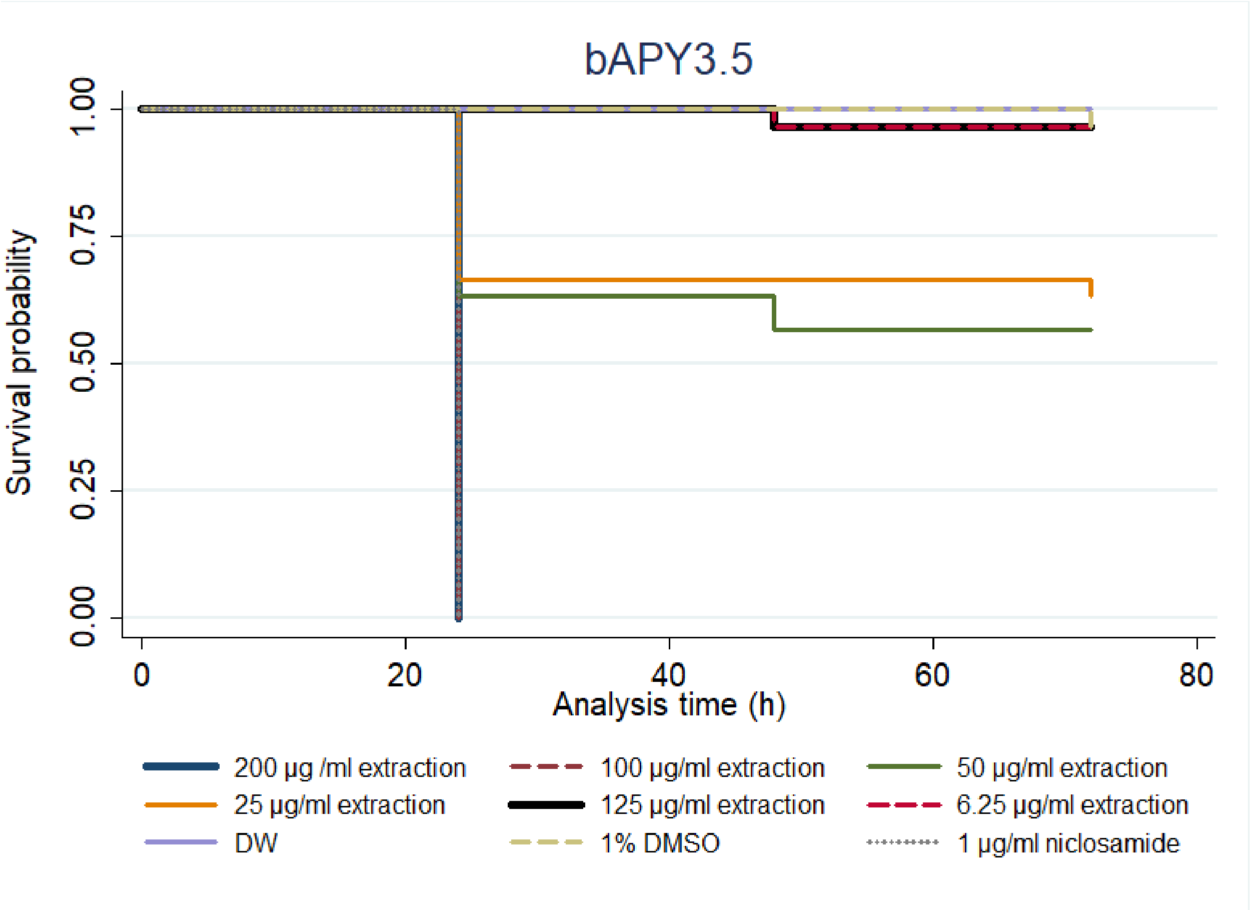


**Fig. S3** Kaplan–Meier overall survival curve comparing the mortality rates of *Indoplanorbis exustus* after exposure to the extract of *Photorhabdus luminescence* subsp. *akhurstii* (bAPY3.5_TH)

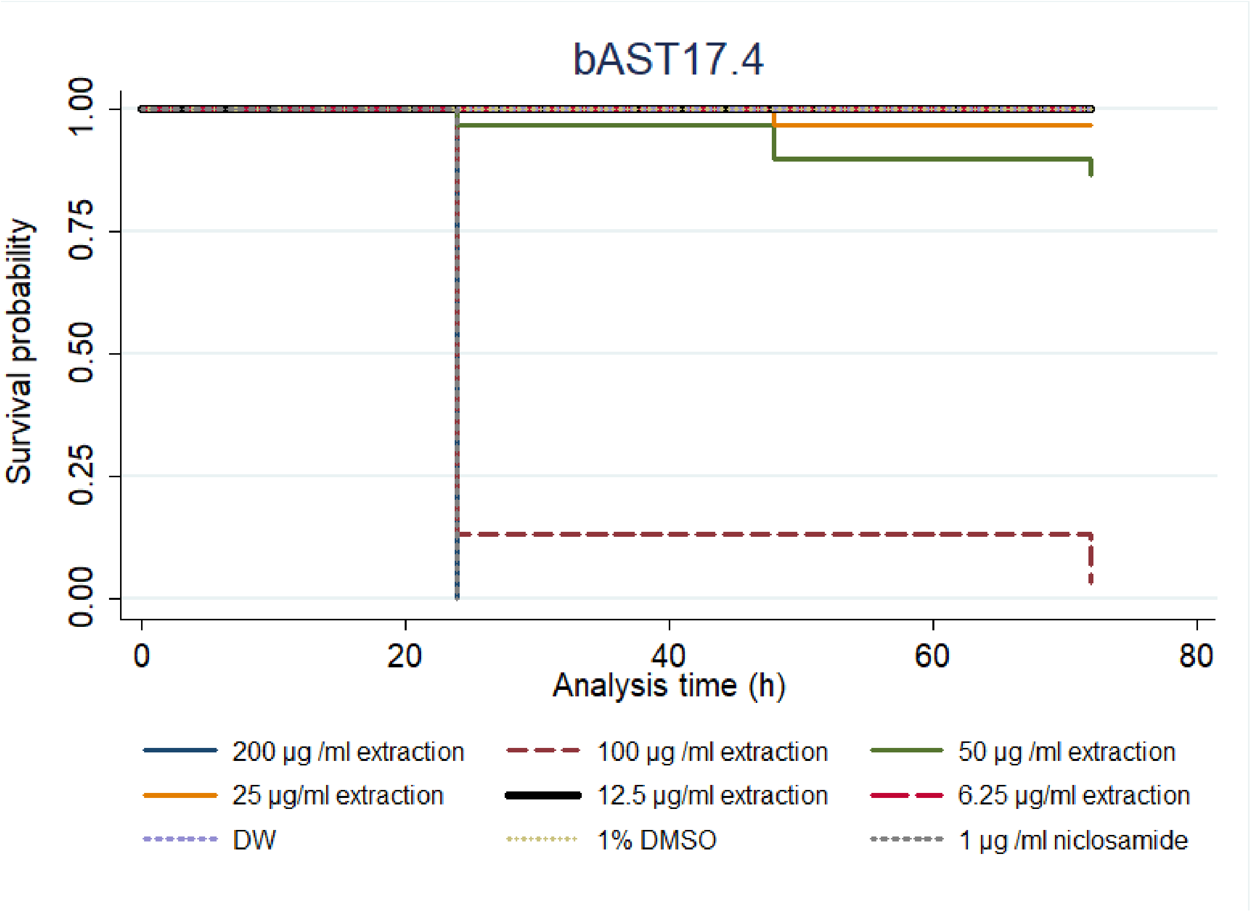


**Fig. S4** Kaplan–Meier overall survival curve comparing the mortality rates of *Indoplanorbis exustus* after exposure to the extract of *Xenorhabdus stockiae* (bAST17.4_TH)

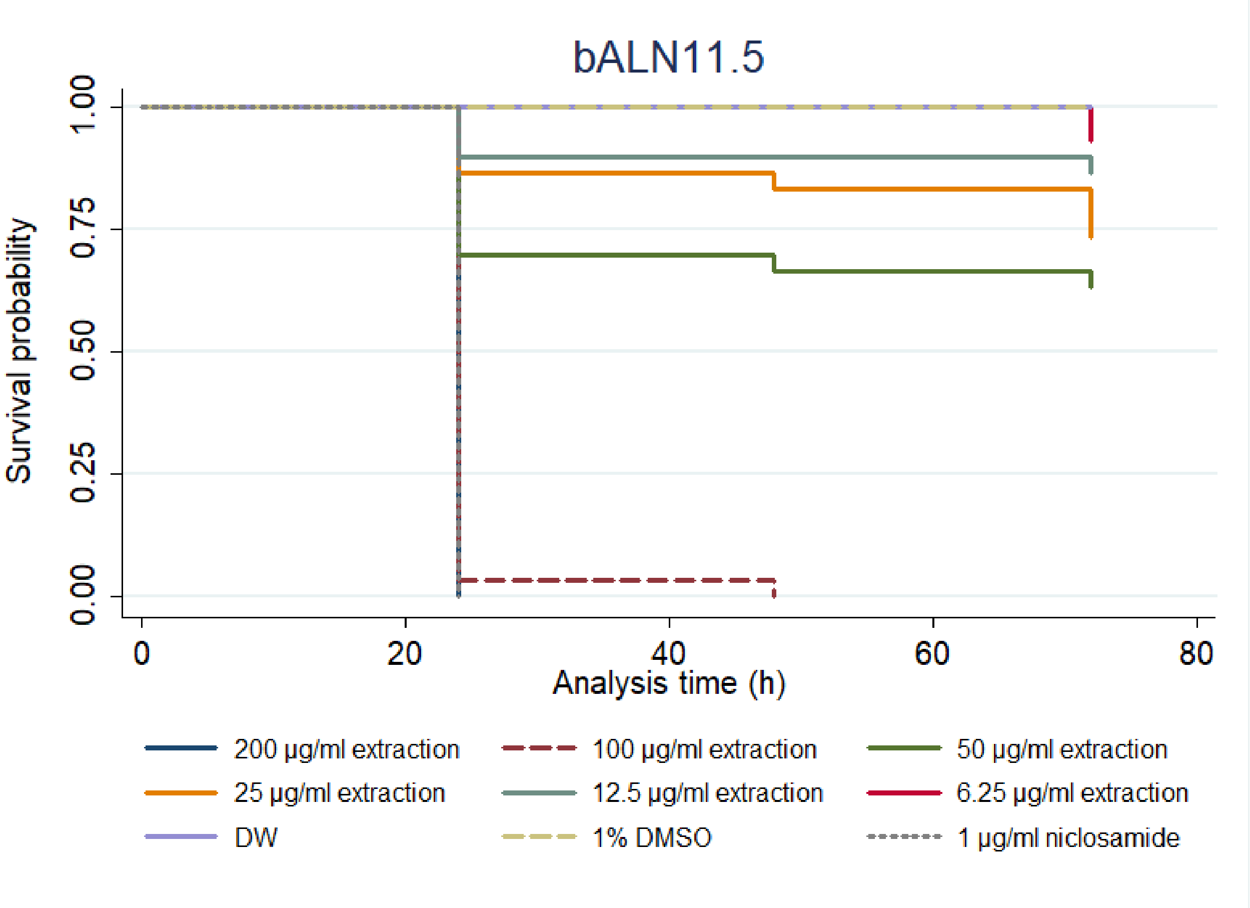


**Fig. S5** Kaplan–Meier overall survival curve comparing the mortality rates of *Radix rubiginosa* after exposure to the extract of *Xenorhabdus ehlersii* (bALN11.5_TH)

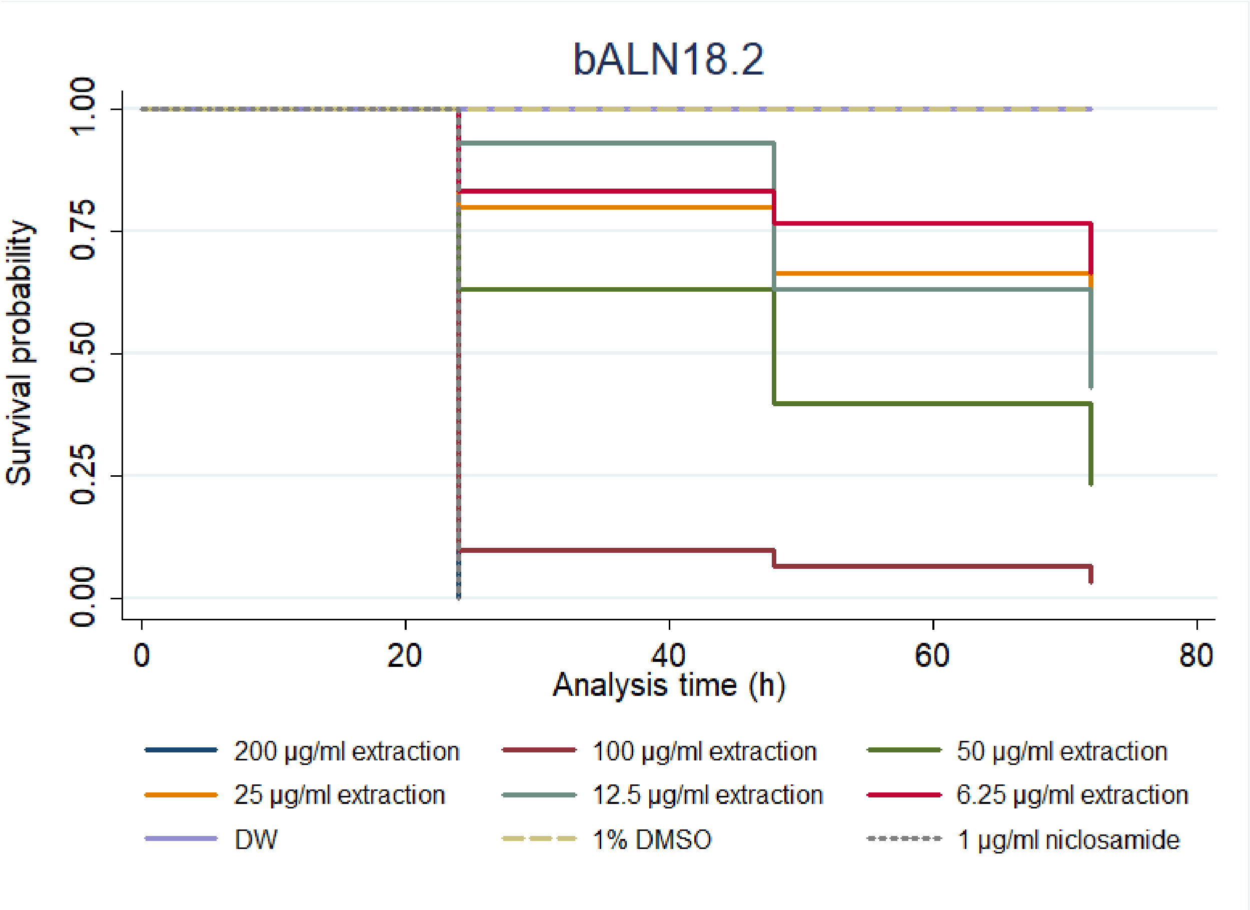


**Fig. S6** Kaplan–Meier overall survival curve comparing the mortality rates of *Radix rubiginosa* after exposure to the extract of *Photorhabdus laumondii* subsp. *laumondii* (bALN18.2_TH)

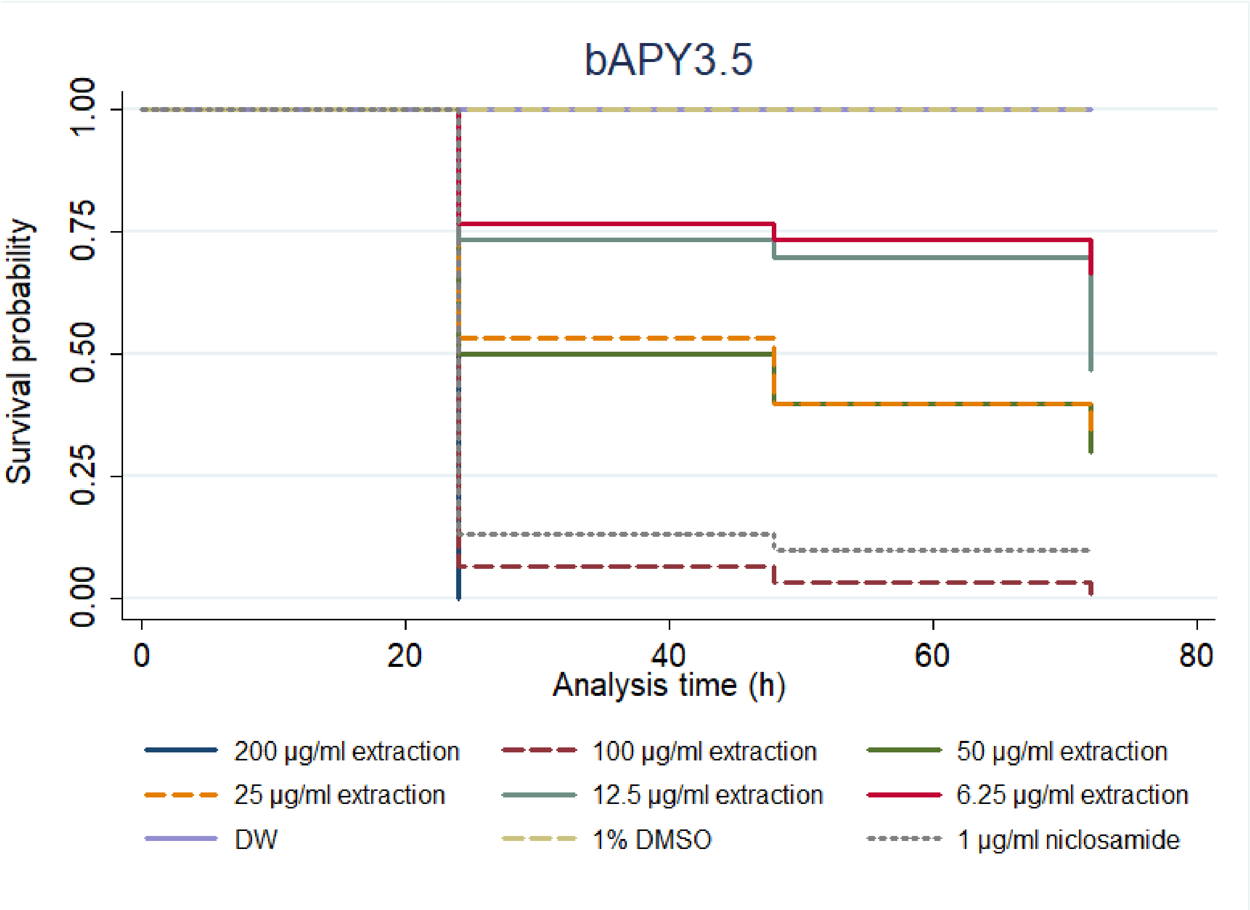


**Fig. S7** Kaplan–Meier overall survival curve comparing the mortality rates of *Radix rubiginosa* after exposure to the extract of *Photorhabdus luminescence* subsp. *akhurstii* (bAPY3.5_TH)

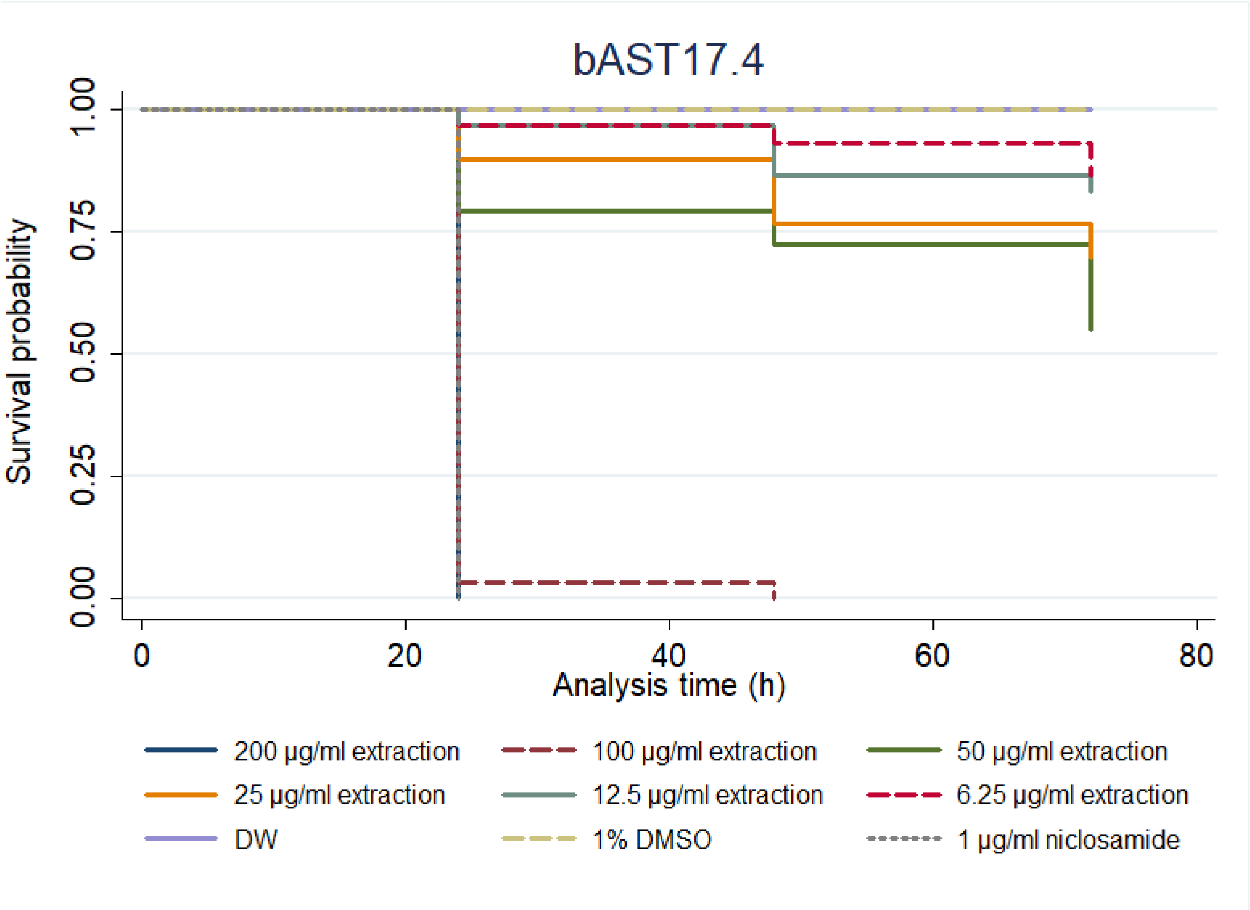


**Fig. S8** Kaplan–Meier overall survival curve comparing the mortality rates of *Radix rubiginosa* after exposure to the extract of *Xenorhabdus stockiae* (bAST17.4_TH)
