# Supplementary material for: Molluscicidal property of symbiotic bacteria associated with entomopathogenic nematodes against Indoplanorbis exustus and Radix rubiginosa, the intermediate hosts of trematode parasites
Source: Parasite Epidemiol Control. 2024 Aug 28;27:e00375. doi: 10.1016/j.parepi.2024.e00375 (PMC11402155; doi:10.1016/j.parepi.2024.e00375)
Supplement: Supplementary file 2 — Supplementary material 2 [file mmc2.pdf]

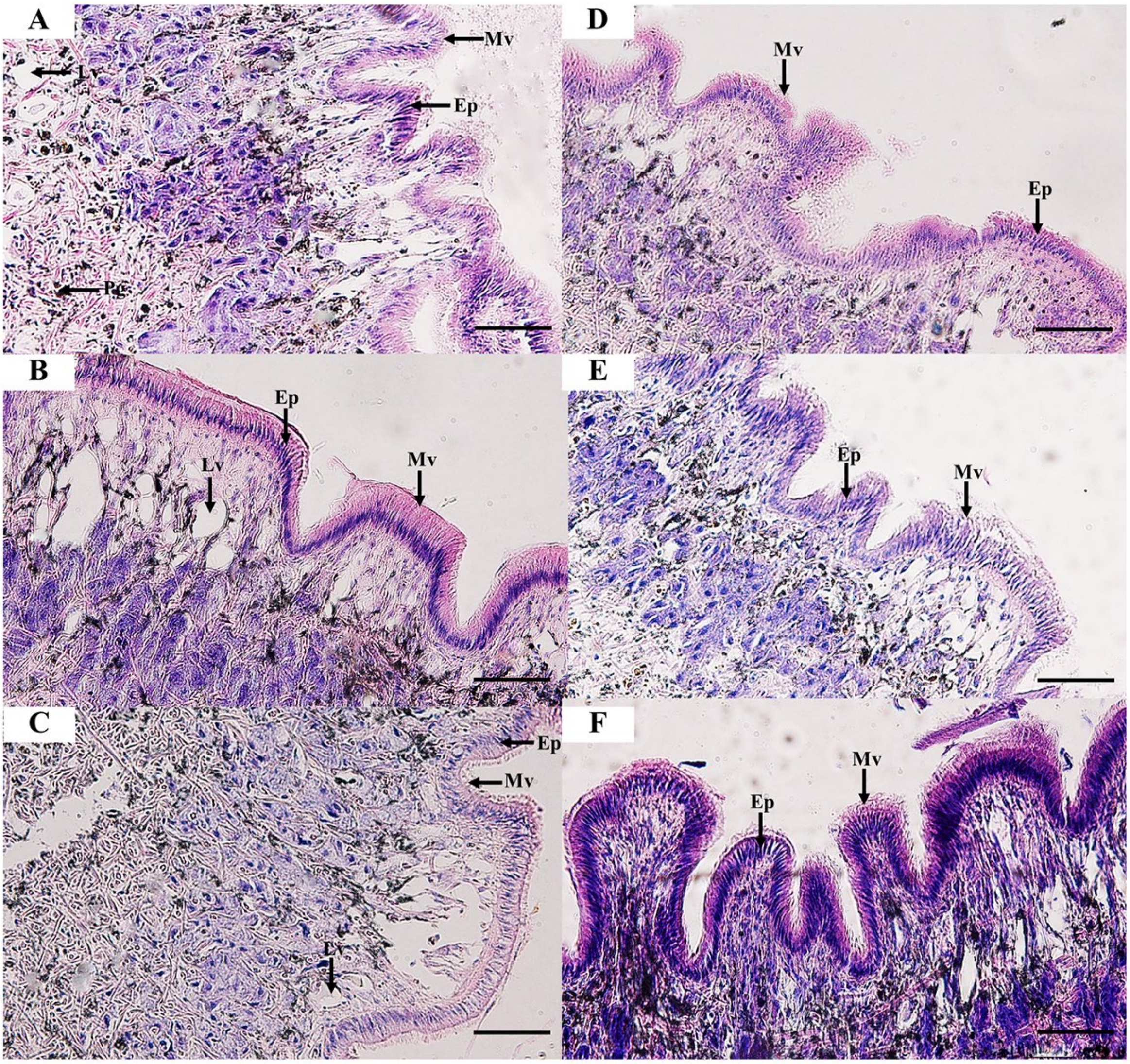

Fig S9. Histological structure of *I. exustus* of epidermis for 3 h in control exposed distilled water (A) and 1% DMSO (B). Snails exposed to *Xenorhabdus stockiae* (bAST17.4_TH) (C), *Xenorhabdus ehlersii* (bALN11.5_TH) (D), *Photorhabdus luminescence* subsp. *akhurstii* (bAPY3.5_TH) (E), and *Photorhabdus laumondii* subsp. *laumondii* (bALN18.2_TH) (F). Ep, Epidermis; Lv, lipid vacuoles; Mv, microvilli; Pg, pigment cells. Scale bar = 50 μm.

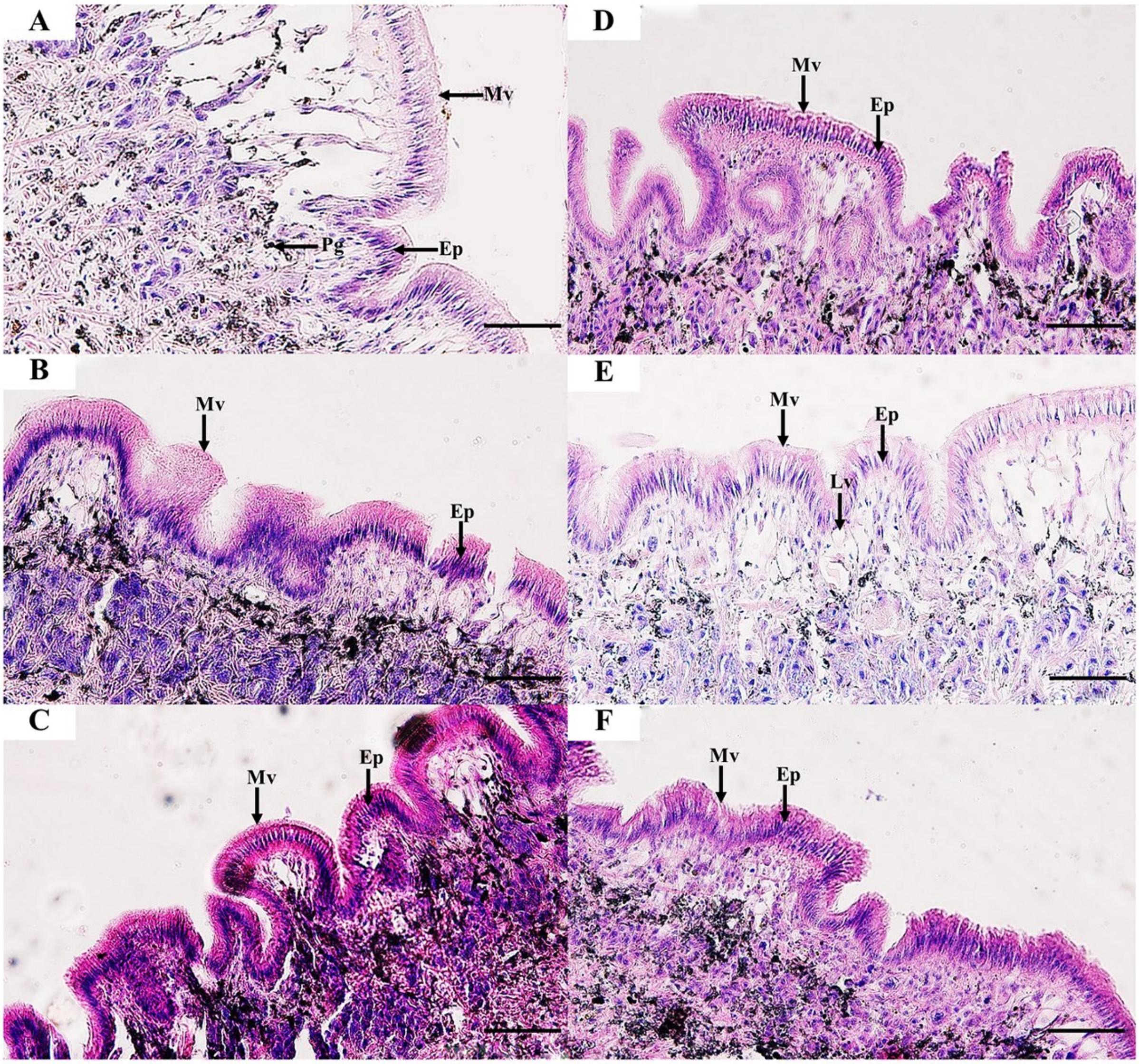

Fig S10. Histological structure of *I. exustus* of epidermis for 6 h in control exposed distilled water (A) and 1% DMSO (B). Snails exposed to *Xenorhabdus stockiae* (bAST17.4_TH) (C), *Xenorhabdus ehlersii* (bALN11.5_TH) (D), *Photorhabdus luminescence* subsp. *akhurstii* (bAPY3.5_TH) (E), and *Photorhabdus laumondii* subsp. *laumondii* (bALN18.2_TH) (F). Ep, Epidermis; Lv, lipid vacuoles; Mv, microvilli; Pg, pigment cells. Scale bar = 50 µm.

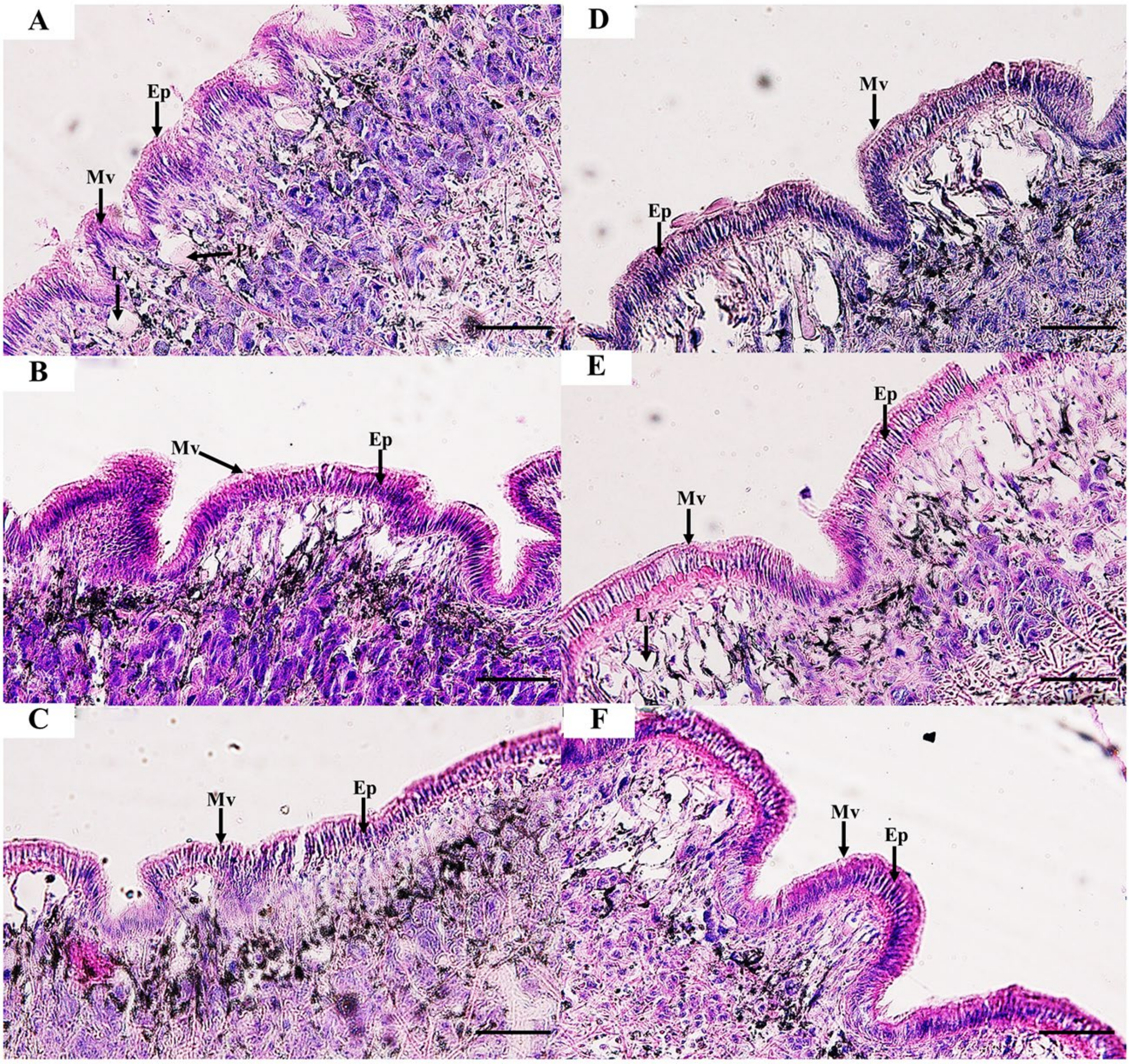

Fig S11. Histological structure of *I. exustus* of epidermis for 12 h in control exposed distilled water (A) and 1% DMSO (B). Snails exposed to *Xenorhabdus stockiae* (bAST17.4_TH) (C), *Xenorhabdus ehlersii* (bALN11.5_TH) (D), *Photorhabdus luminescence* subsp. *akhurstii* (bAPY3.5_TH) (E), and *Photorhabdus laumondii* subsp. *laumondii* (bALN18.2_TH) (F). Ep, Epidermis; Lv, lipid vacuoles; Mv, microvilli; Pg, pigment cells. Scale bar = 50 µm.

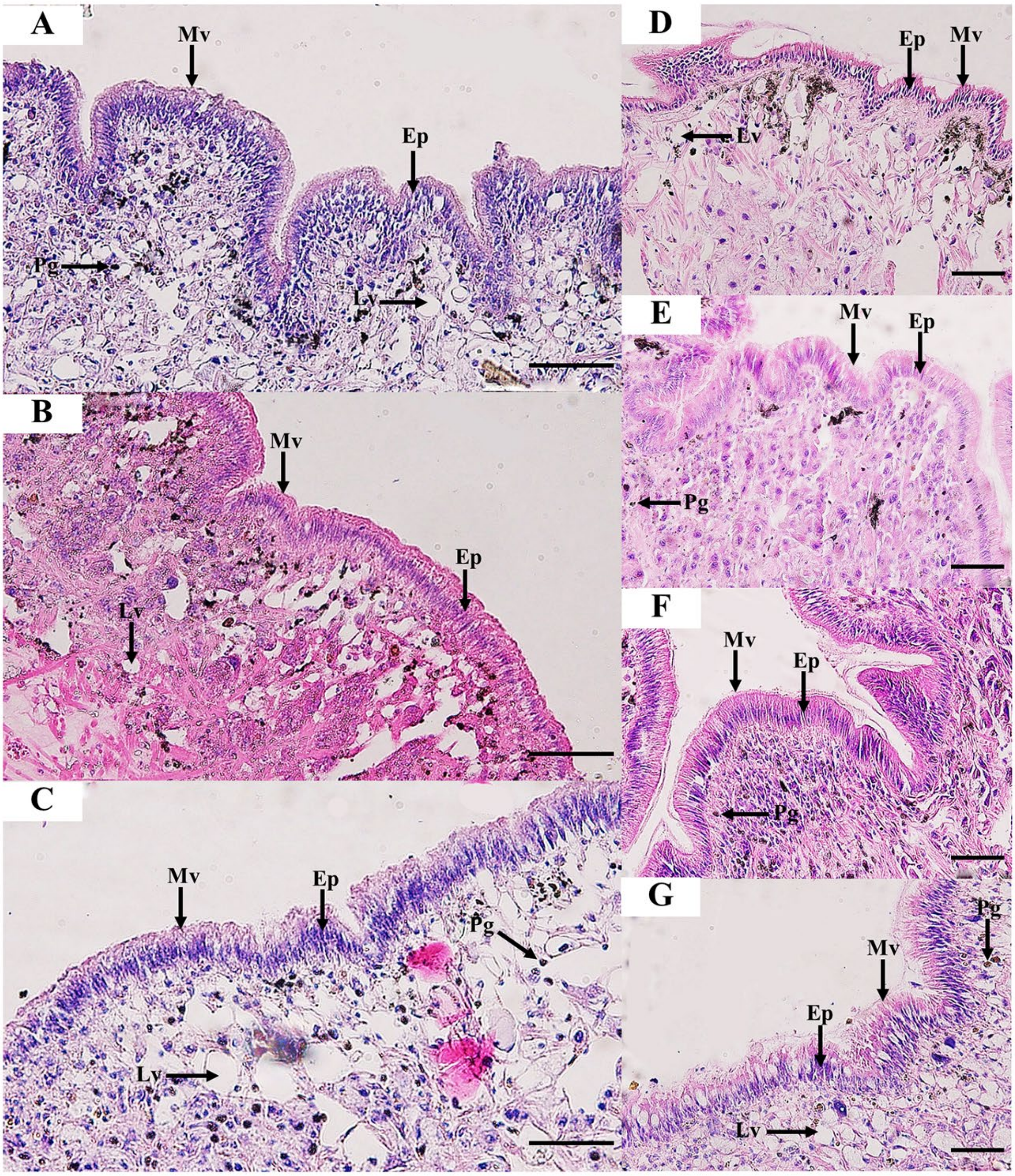

Fig S12. Histological structure of *R. rubiginosa* of epidermis for 3 h in control exposed distilled water (A), 1% DMSO (B), and 1% Niclosamide (C). Snails exposed to *Xenorhabdus stockiae* (bAST17.4_TH) (D), *Xenorhabdus ehlersii* (bALN11.5_TH) (E), *Photorhabdus luminescence* subsp. *akhurstii* (bAPY3.5_TH) (F), and *Photorhabdus laumondii* subsp. *laumondii* (bALN18.2_TH) (G). Ep, Epidermis; Lv, lipid vacuoles; Mv, microvilli; Pg, pigment cells. Scale bar = 50 µm.

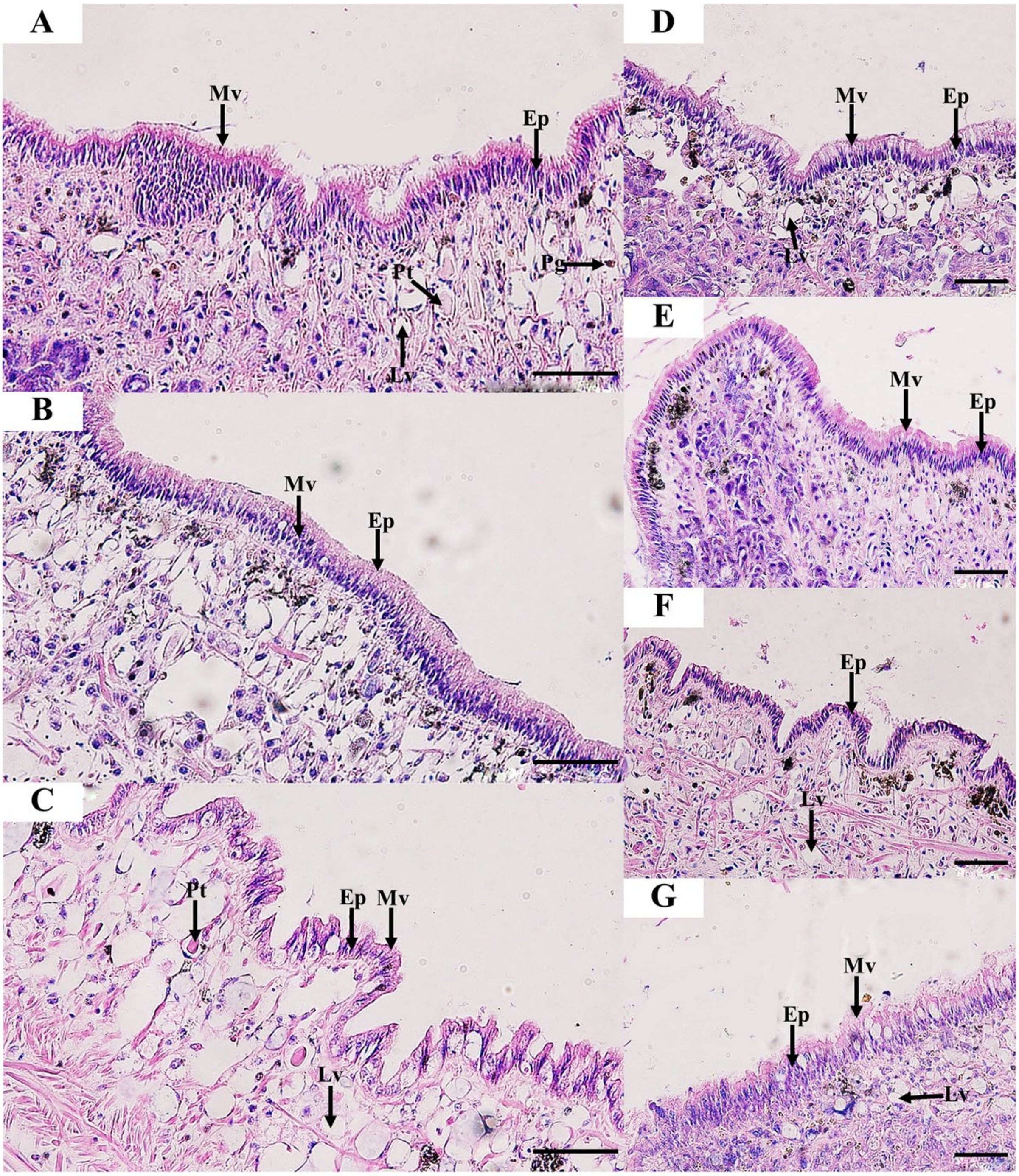

Fig S13. Histological structure of *R. rubiginosa* of epidermis for 6 h in control exposed distilled water (A), 1% DMSO (B), and 1% Niclosamide (C). Snails exposed to *Xenorhabdus stockiae* (bAST17.4_TH) (D), *Xenorhabdus ehlersii* (bALN11.5_TH) (E), *Photorhabdus luminescence* subsp. *akhurstii* (bAPY3.5_TH) (F), and *Photorhabdus laumondii* subsp. *laumondii* (bALN18.2_TH) (G). Ep, Epidermis; Lv, lipid vacuoles; Mv, microvilli; Pg, pigment cells. Scale bar = 50 µm.

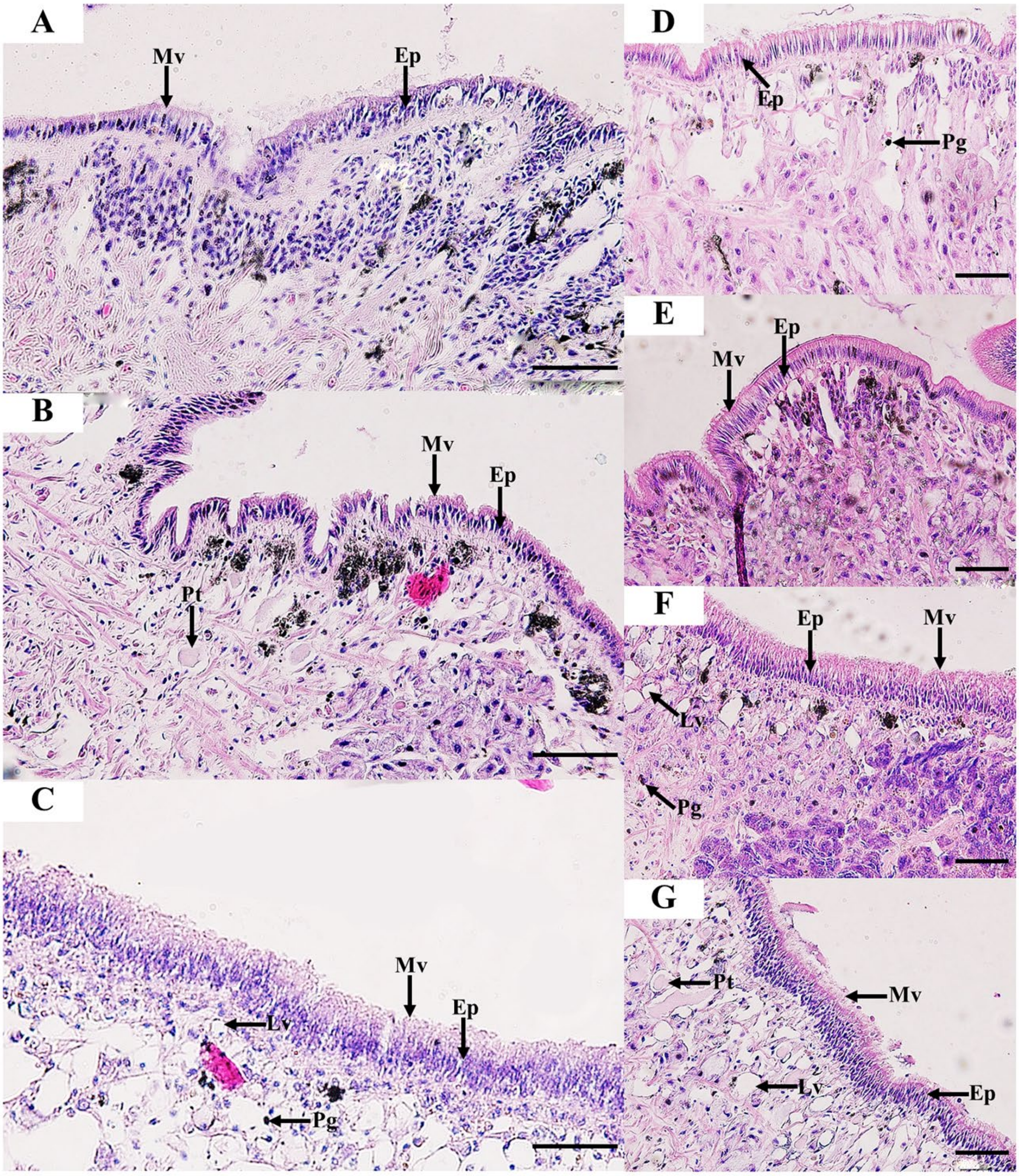

Fig S14. Histological structure of *R. rubiginosa* of epidermis for 12 h in control exposed distilled water (A), 1% DMSO (B), and 1% Niclosamide (C). Snails exposed to *Xenorhabdus stockiae* (bAST17.4_TH) (D), *Xenorhabdus ehlersii* (bALN11.5_TH) (E), *Photorhabdus luminescence* subsp. *akhurstii* (bAPY3.5_TH) (F), and *Photorhabdus laumondii* subsp. *laumondii* (bALN18.2_TH) (G). Ep, Epidermis; Lv, lipid vacuoles; Mv, microvilli; Pg, pigment cells. Scale bar = 50 µm.

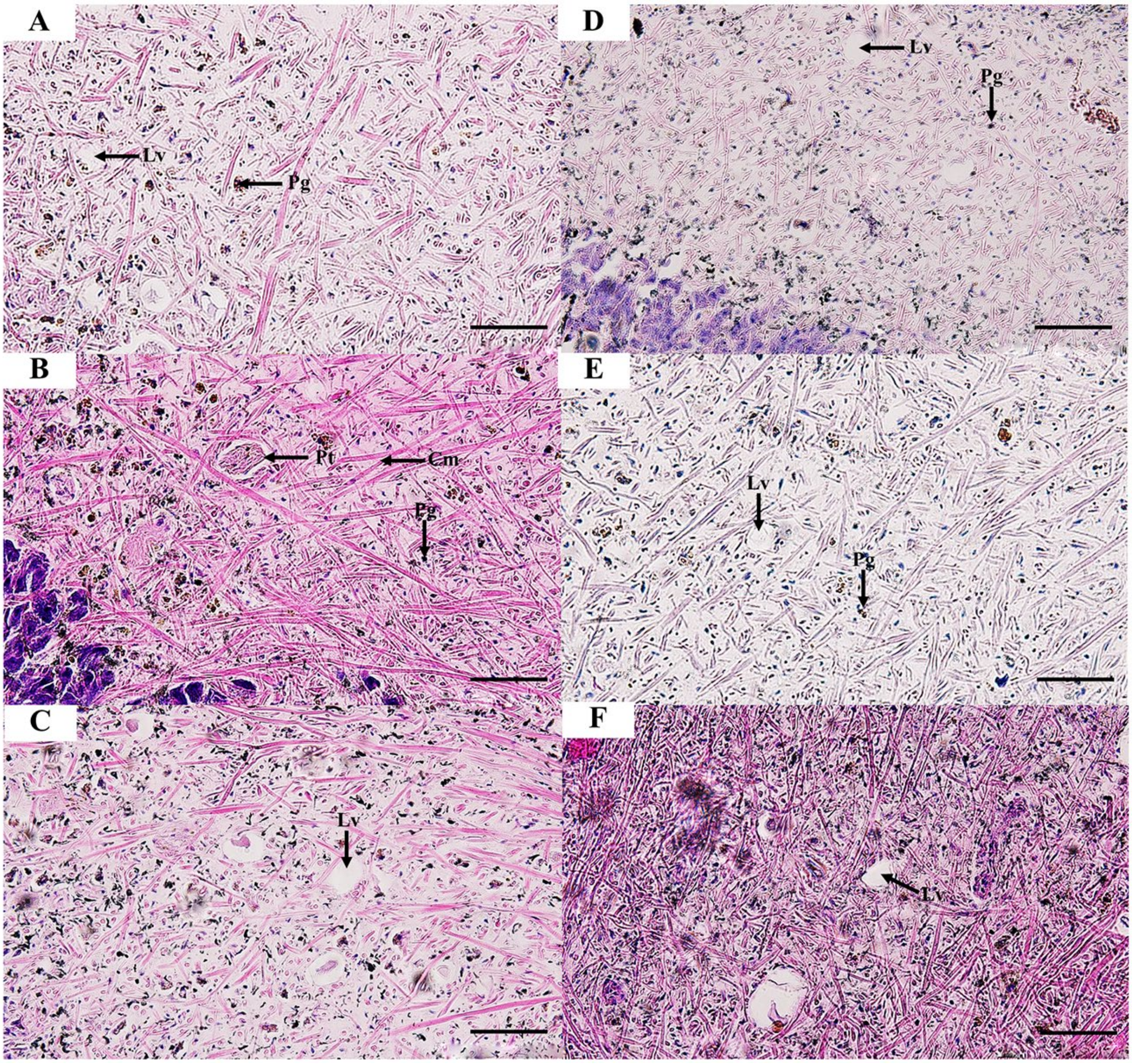

Fig S15. Histological structure of *I. exustus* of foot tissue for 3 h in control exposed distilled water (A) and 1% DMSO (B). Snails exposed to *Xenorhabdus stockiae* (bAST17.4_TH) (C), *Xenorhabdus ehlersii* (bALN11.5_TH) (D), *Photorhabdus luminescence* subsp. *akhurstii* (bAPY3.5_TH) (E), and *Photorhabdus laumondii* subsp. *laumondii* (bALN18.2_TH) (F). Cm, columnar muscle fibers; Lv, lipid vacuoles; Pg, pigment cells; Pt, protein cells. Scale bar = 50 μm.

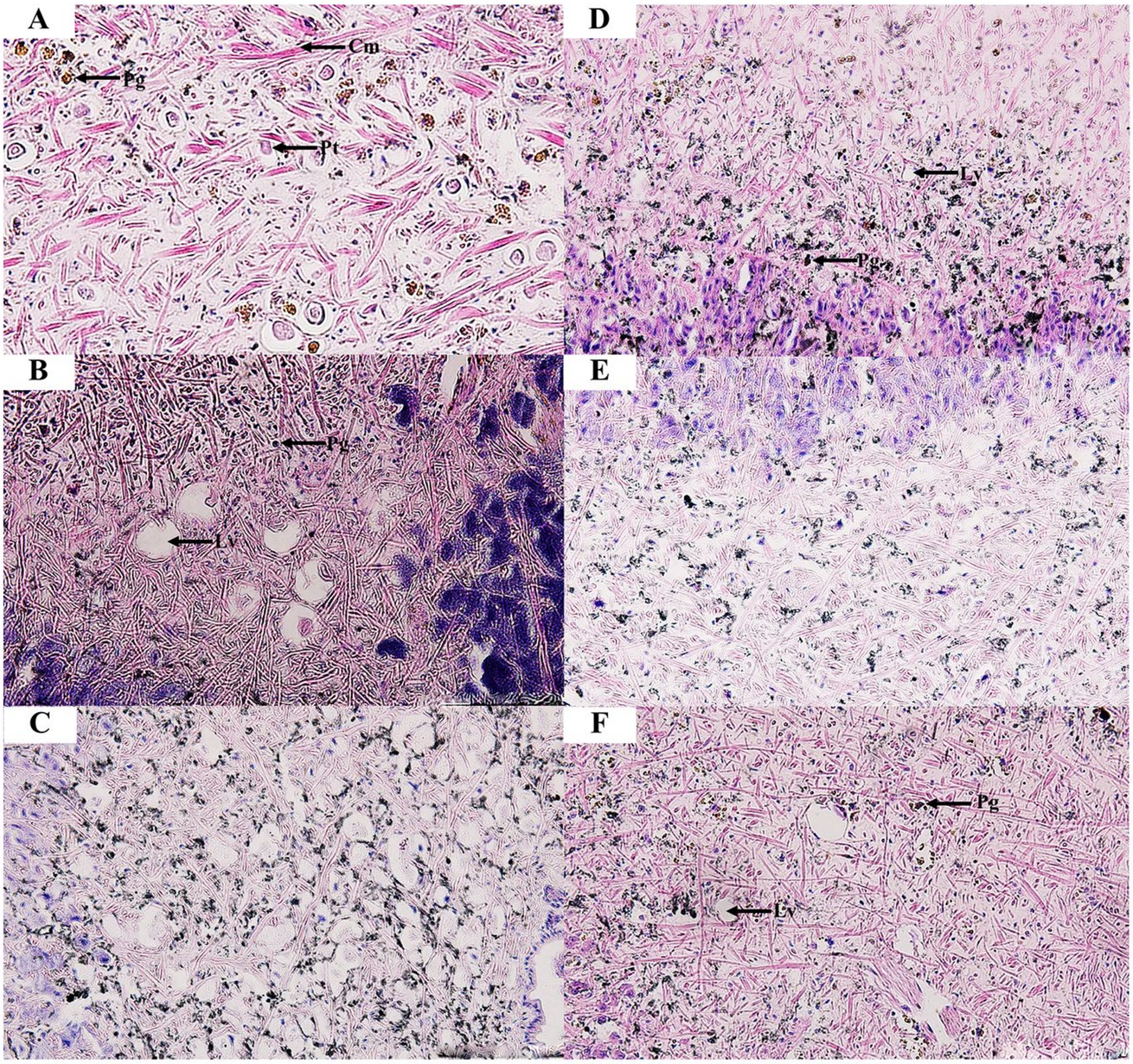

Fig S16. Histological structure of *I. exustus* of foot tissue for 6 h in control exposed distilled water (A) and 1% DMSO (B). Snails exposed to *Xenorhabdus stockiae* (bAST17.4_TH) (C), *Xenorhabdus ehlersii* (bALN11.5_TH) (D), *Photorhabdus luminescence* subsp. *akhurstii* (bAPY3.5_TH) (E), and *Photorhabdus laumondii* subsp. *laumondii* (bALN18.2_TH) (F). Cm, columnar muscle fibers; Lv, lipid vacuoles; Pg, pigment cells; Pt, protein cells. Scale bar = 50 μm.

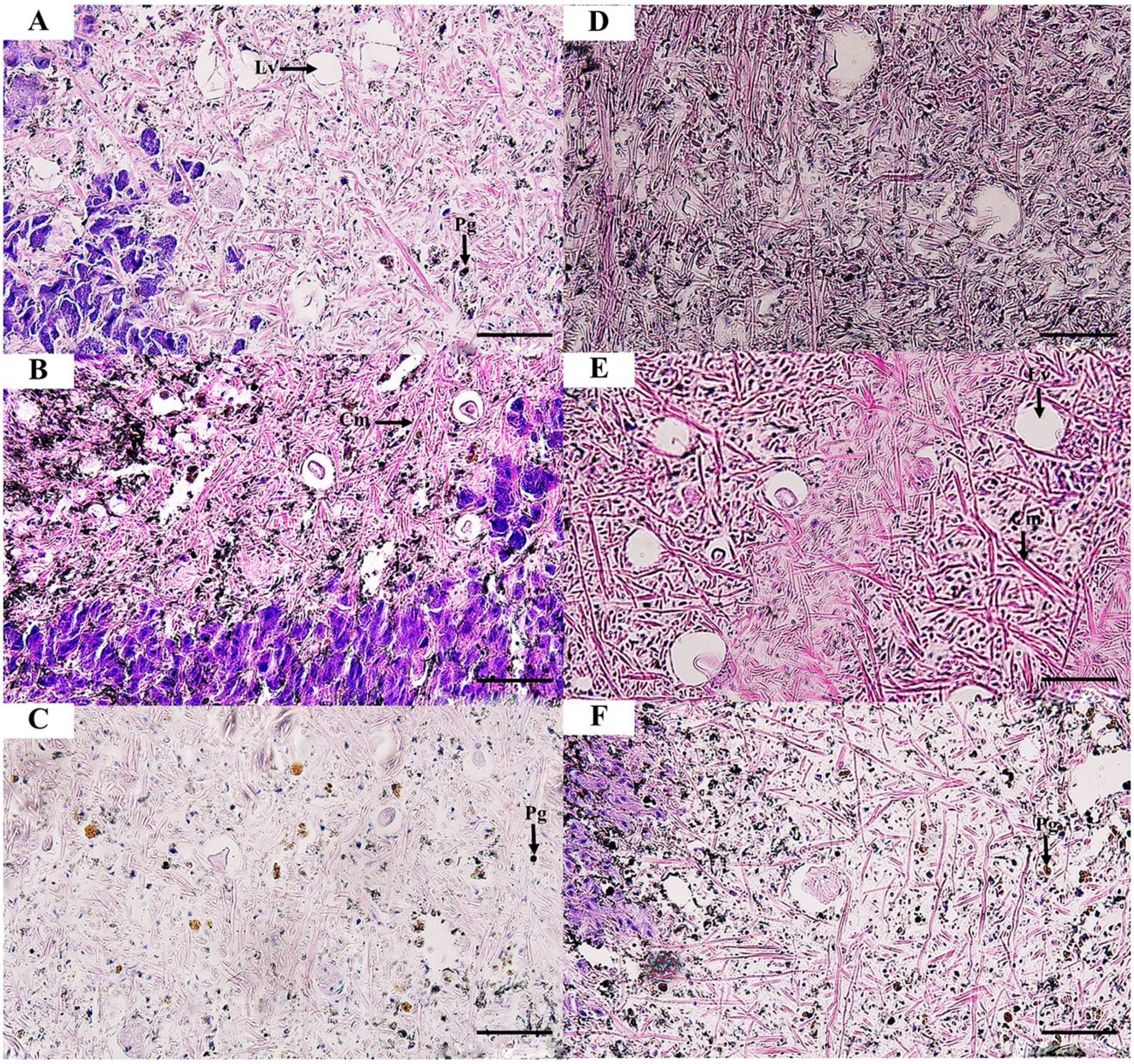

Fig S17. Histological structure of *I. exustus* of foot tissue for 12 h in control exposed distilled water (A) and 1% DMSO (B). Snails exposed to *Xenorhabdus stockiae* (bAST17.4_TH) (C), *Xenorhabdus ehlersii* (bALN11.5_TH) (D), *Photorhabdus luminescence* subsp. *akhurstii* (bAPY3.5_TH) (E), and *Photorhabdus laumondii* subsp. *laumondii* (bALN18.2_TH) (F). Cm, columnar muscle fibers; Lv, lipid vacuoles; Pg, pigment cells; Pt, protein cells. Scale bar = 50 μm.

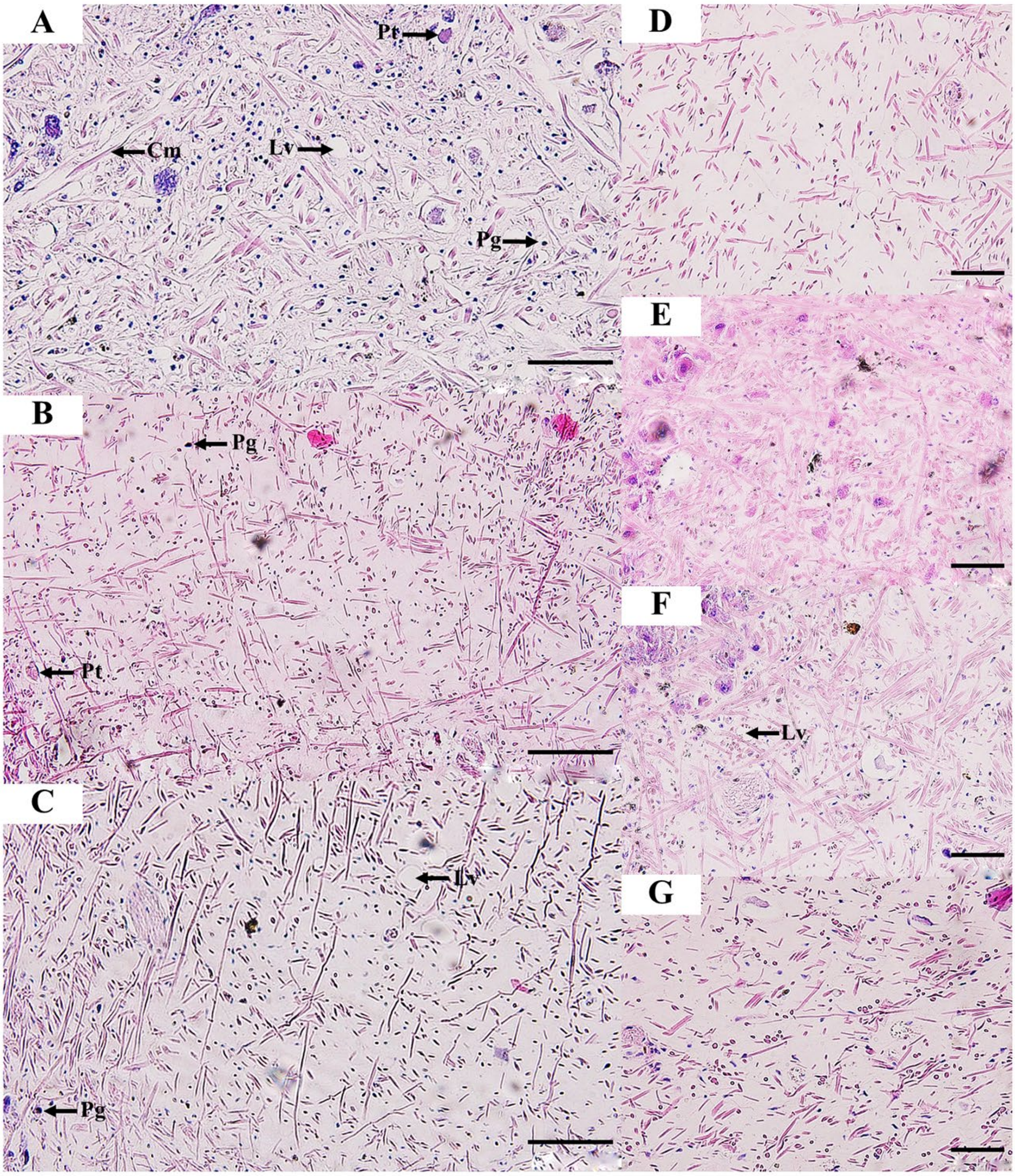

Fig S18. Histological structure of *R. rubiginosa* of foot tissue for 3 h in control exposed distilled water (A), 1% DMSO (B), and 1% Niclosamide (C). Snails exposed to *Xenorhabdus stockiae* (bAST17.4_TH) (D), *Xenorhabdus ehlersii* (bALN11.5_TH) (E), *Photorhabdus luminescence* subsp. *akhurstii* (bAPY3.5_TH) (F), and *Photorhabdus laumondii* subsp. *laumondii* (bALN18.2_TH) (G). Cm, columnar muscle fibers; Lv, lipid vacuoles; Pg, pigment cells; Pt, protein cells. Scale bar = 50 µm.

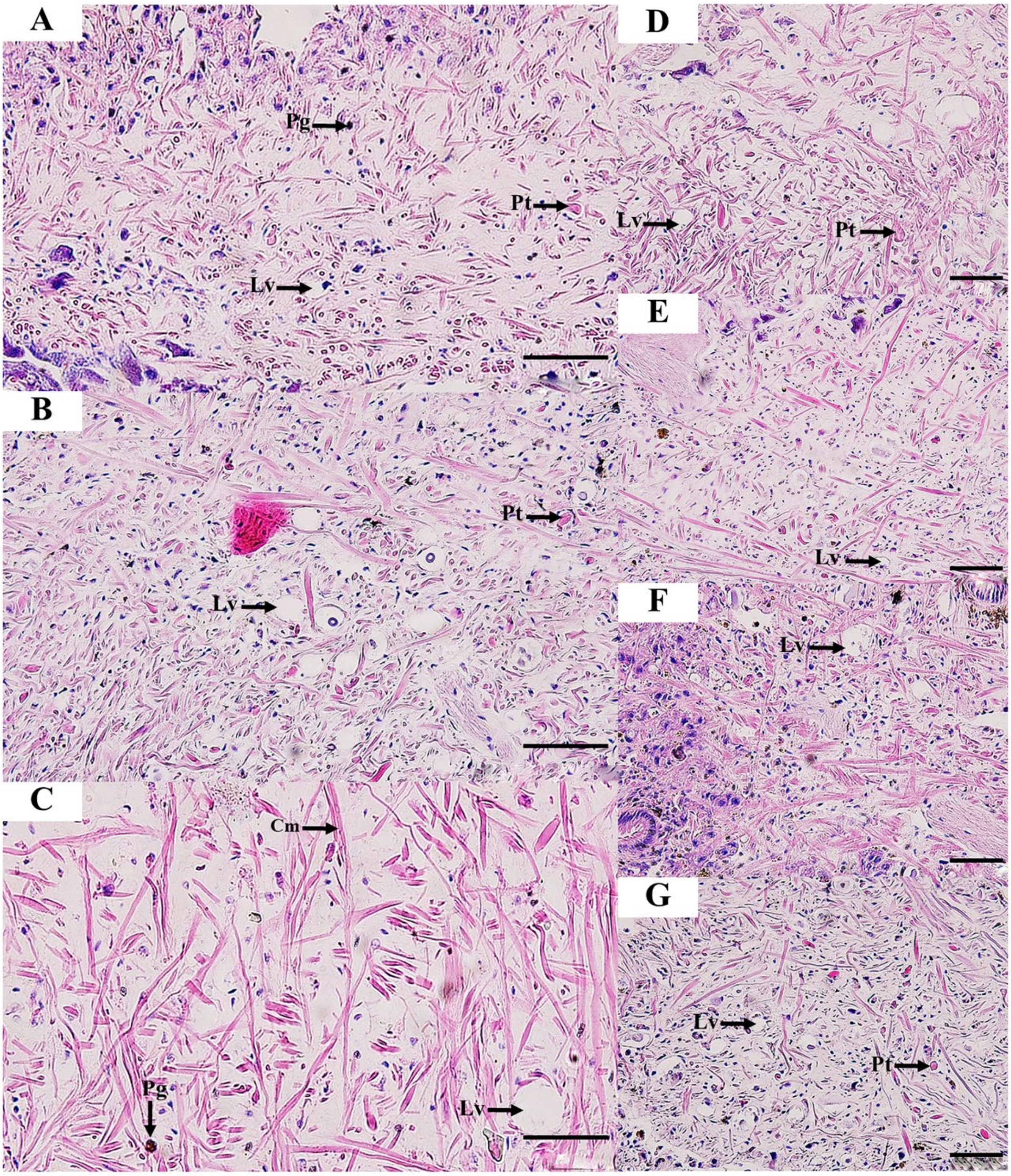

Fig S19. Histological structure of *R. rubiginosa* of foot tissue for 6 h in control exposed distilled water (A), 1% DMSO (B), and 1% Niclosamide (C). Snails exposed to *Xenorhabdus stockiae* (bAST17.4_TH) (D), *Xenorhabdus ehlersii* (bALN11.5_TH) (E), *Photorhabdus luminescence* subsp. *akhurstii* (bAPY3.5_TH) (F), and *Photorhabdus laumondii* subsp. *laumondii* (bALN18.2_TH) (G). Cm, columnar muscle fibers; Lv, lipid vacuoles; Pg, pigment cells; Pt, protein cells. Scale bar = 50 µm.

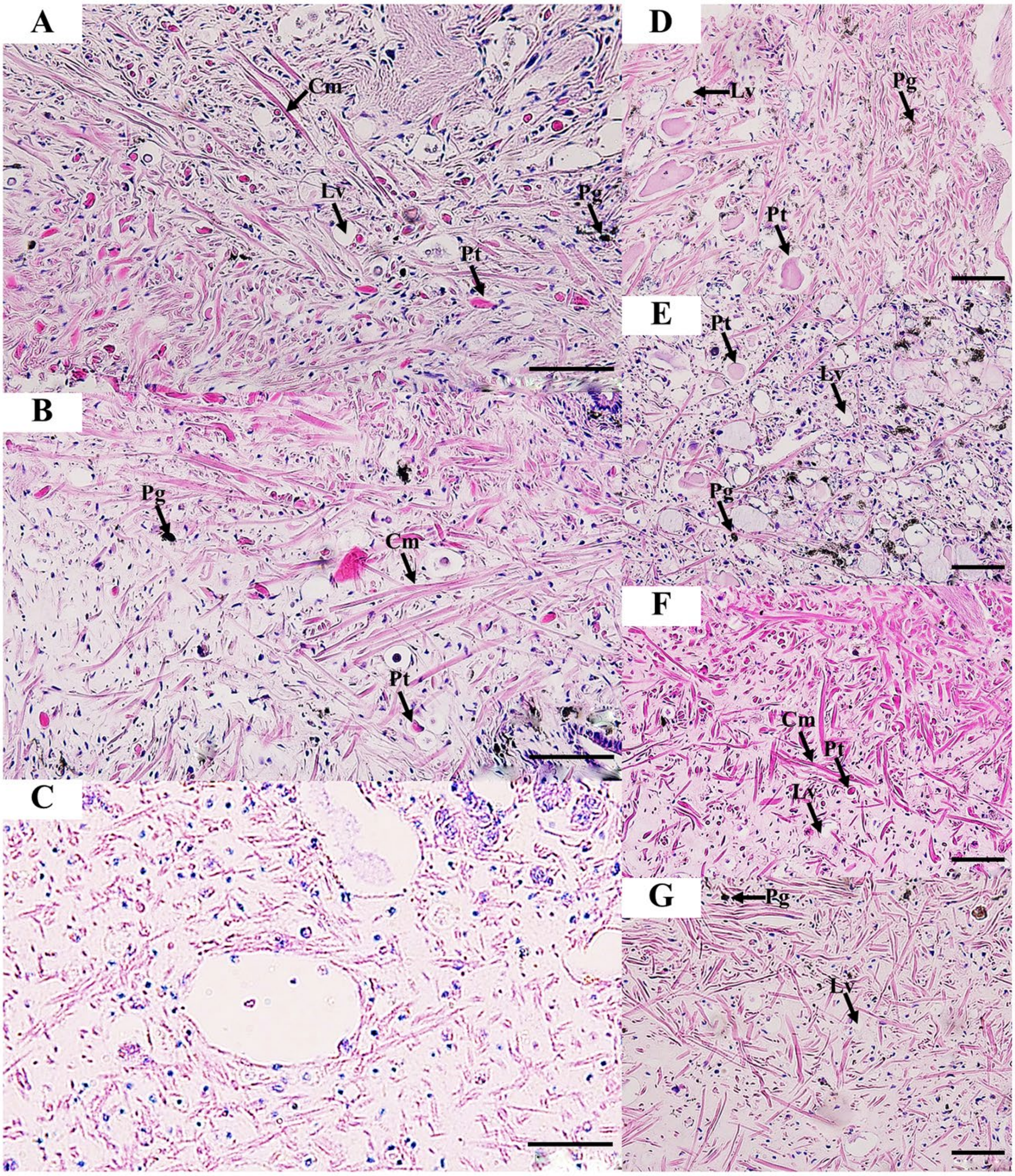

Fig S20. Histological structure of *R. rubiginosa* of foot tissue for 12 h in control exposed distilled water (A), 1% DMSO (B), and 1% Niclosamide (C). Snails exposed to *Xenorhabdus stockiae* (bAST17.4_TH) (D), *Xenorhabdus ehlersii* (bALN11.5_TH) (E), *Photorhabdus luminescence* subsp. *akhurstii* (bAPY3.5_TH) (F), and *Photorhabdus laumondii* subsp. *laumondii* (bALN18.2_TH) (G). Cm, columnar muscle fibers; Lv, lipid vacuoles; Pg, pigment cells; Pt, protein cells. Scale bar = 50 µm.
